# Comparative transcriptome and metabolome analysis of *Ostrinia furnacalis* female adults under UV-A exposure

**DOI:** 10.1038/s41598-021-86269-0

**Published:** 2021-03-24

**Authors:** Li Su, Changli Yang, Jianyu Meng, Lv Zhou, Changyu Zhang

**Affiliations:** 1 Institute of Entomology, Provincial Key Laboratory for Agricultural Pest Management of Mountainous Regions, Guizhou University, Guiyang, 550025 People’s Republic of China; 2 Guizhou Tobacco Science Research Institute, Guiyang, 550081 People’s Republic of China

**Keywords:** Transcription, Transcriptomics

## Abstract

Ultraviolet A (UV-A) radiation is a significant environmental factor that causes photoreceptor damage, apoptosis, and oxidative stress in insects. *Ostrinia furnacalis* is an important pest of corn. To understand the adaptation mechanisms of insect response to UV-A exposure, this study revealed differentially expressed genes (DEGs) and differently expressed metabolites (DEMs) in *O. furnacalis* under UV-A exposure. Three complementary DNA libraries were constructed from *O. furnacalis* adult females (CK, UV1h, and UV2h), and 50,106 expressed genes were obtained through Illumina sequencing. Of these, 157 and 637 DEGs were detected in UV1h and UV2h after UV-A exposure for 1 and 2 h, respectively, compared to CK, with 103 and 444 upregulated and 54 and 193 downregulated genes, respectively. Forty four DEGs were detected in UV2h compared to UV1h. Comparative transcriptome analysis between UV-treated and control groups revealed signal transduction, detoxification and stress response, immune defense, and antioxidative system involvement. Metabolomics analysis showed that 181 (UV1h vs. CK), 111 (UV2h vs. CK), and 34 (UV2h vs. UV1h) DEMs were obtained in positive ion mode, while 135 (UV1h vs. CK), 93 (UV2h vs. CK), and 36 (UV2h vs. UV1h) DEMs were obtained in negative ion mode. Moreover, UV-A exposure disturbed amino acid, sugar, and lipid metabolism. These findings provide insight for further studies on how insects protect themselves under UV-A stress.

## Introduction

Solar ultraviolet (UV) radiation, including UV-A (315–400 nm), UV-B (280–315 nm), and UV-C (200–280 nm), is a severe environmental factor that affects the ecosystem and every living organism^1^. Because of ozone layer depletion in the past few decades, solar UV radiation levels have gradually increased^2^. UV-A constitutes ~ 95% of solar UV radiation reaching the surface of the earth^3^. UV-A is used by insects to acquire visual cues for foraging and selecting mates^4^. However, UV-A has many direct and indirect negative effects, such as oxidative stress, photoreceptor damage, and apoptosis, in almost all organisms, including insects^5,6^. UV-A alters juvenile hormone (JH) metabolism and decreases the life span of *Helicoverpa armigera* adults^7,8^. It can also damage the photoreceptive cells, lipids, and DNA in the compound eyes of insects^9^. In addition, UV-A causes reactive oxygen species (ROS) formation in insects and disturbs the functional activity of antioxidant enzymes^10–12^. Insects have developed various protective mechanisms against harmful UV-A, such as strengthening the reproductive capacity as a trade-off of energy apportion between reproduction and longevity under UV-A stress^7^, regulating antioxidant enzymes activity^13^, activating the mitogen-activated protein kinase (MAPK) signaling pathway to respond to UV-A stress^14^, and regulating the expression of stress-responsive genes, such as cytochrome P450 monooxygenase (*p450s*) and heat shock protein (*hsp*), which might participate in decreasing UV-induced ecological challenges^15,16^. Both injurious and protective mechanisms in insects are complex, network functions rather than a single enzyme or protein function. The adaptive mechanism of insects to UV-A exposure is multifaceted and involves various genetic and metabolic factors responsible for resistance against UV-A stress, such as gene regulation and metabolite expression.

Transcriptomics is an effective tool for analyzing the function of some key genes and compare gene expression under different stress conditions^17^. Metabonomics is a useful tool for identifying metabolites and explaining changes in them in a biological system under different stress conditions^18^. Numerous studies have reported transcriptome and metabolome analysis of insects under various environmental stresses, such as solar UV radiation, temperature, fungi, viruses, and hypoxia^16,19–24^.

The Asian corn borer *Ostrinia furnacalis* (Guenée) (Lepidoptera: Pyralidae) is an important global agricultural pest that frequently causes serious economic damage to corn, cotton, millet, and sorghum^26,27^. Adults have strong phototaxis and are highly sensitive to UV-A light^28^. UV-A is a common light source for light traps. Previous studies demonstrated that the blacklight trap technology is an effective field forecasting means and an important technology used to control lepidopteran insects ^25,73,74^. UV-A is used as black light for forecasting and controlling *O. furnacalis* and directly affects the behavior and visual physiology of *O. furnacalis*^29^. Studies also have reported the effects of UV-A on the proteome, reproductive system, and signal pathway proteins of *O. furnacalis*^30–32^. Therefore, *O. furnacalis* might have specific adaptive strategies for responding to UV-A stress in order to better adapt to environmental stresses. This study investigated the adaptation mechanisms of adult female *O. furnacalis*’s UV-A exposure response after 0, 1, and 2 h. High-throughput RNA sequencing (RNA-seq) and liquid chromatography–mass spectrometry (LC–MS)-based metabolomics analysis was performed to explore the mechanism underlying UV-A stress response on the basis of gene expression and metabolite patterns in *O. furnacalis*.

## Materials and methods

### Insect rearing and UV-A treatment

*O. furnacalis* were collected from corn fields in Puding County (26° 32′ N, 105° 75′ E), Guizhou Province, China, and reared in climate cabinets in 70% ± 10% relative humidity and a 14 h/10 h light/dark cycle at 27  ± 1 °C. The larvae were reared on artificial diets, as previously described^33^. Adult insects were provided a 10% honey water solution.

Next, 3-day-old females were irradiated with a UV-A lamp (320–400 nm; Nanjing Huaqiang Electronic Engineering Co. Ltd., China). The light intensity was ~ 300 μW/cm^2^, and the temperature was maintained at 26 °C. After preadaptation of a 2 h scotophase, three groups of nine 3-day-old females each were treated with UV-A for 0 (CK; control), 1 (UV1h), and 2 h (UV2h). The two treatment groups were replicated three times, while the control group was replicated eight times. All insects were collected and stored at − 80 °C until transcriptomic and metabolomics analysis.

## Transcriptomic analysis

### RNA extraction, cDNA library construction, and Illumina sequencing

The method described by Nayak et al*.*, 2020^70^ was used to prepare libraries. Total RNA was extracted from 3-day-old *O. furnacalis* females using TRIzol reagent (Invitrogen Carlsbad, CA, USA) according to the manufacturer’s instructions. The RNA obtained was treated with DNase I (Invitrogen), and magnetic beads with oligo (dT) were used to isolate poly(A) + messenger RNA (mRNA) and sheared into short fragments using a fragmentation buffer. Using the mRNA as a template, first-strand complementary DNA (cDNA) was synthesized using random hexamer primers, and then adding a buffer, deoxynucleoside triphosphates (dNTPs), DNA polymerase I, and RNase H, second-strand cDNA was synthesized and purified using the AMPure XP system (Beckman Coulter, Beverly, MA, USA). Next, the purified double-stranded cDNA was subjected to an end repair process, and poly(A) was added, and suitable fragments were selected and enriched by polymerase chain reaction (PCR) to construct three cDNA libraries. The cDNA library quality was assessed using the Agilent Bioanalyzer 2100 system (Agilent Technologies, Santa Clara, CA, USA), which was sequenced by Novogene (Beijing, China) on the Illumina Hiseq platform (Illumina, San Diego, CA, USA).

### De novo assembly and functional gene annotation

Before assembly, we removed the adaptor, *N* < 10%, and low-quality sequences from raw data to obtain clean reads, which were then de novo*-*assembled using Trinity software^34^ (http://trinityrnaseq.sourceforge.net/) to produce nonredundant unigenes without a reference genome. Subsequently, the unigenes assembled were analyzed and annotated using the Basic Local Alignment Search Tool with the nucleotide sequence databases (Nt), nonredundant protein database (Nr), Swiss-Prot, EuKaryotic Orthologous Groups (KOG), and the Kyoto Encyclopedia of Genes and Genomes (KEGG) (*e*-value < 10 − 5). Finally, functional Gene Ontology (GO) annotations were obtained for All-unigenes using the Blast 2 GO program on the basis of the biological process, molecular function, and cellular component categories^35,36^.

### Differential gene expression and phylogenetic analysis

To determine the differential unigene abundance, independent alignments of short reads from three cDNA libraries were performed against the set of *O. furnacalis* in response to UV treatment unigenes using Blat software. Unigene transcripts were quantified using the RSEM tool^37^. Quantitative results were expressed in fragments per kilobase of transcript sequence per million mapped reads (FPKM)^38^ as follows:

$${\text{FPKM }}\left( A \right)\, = \,\left( {{1},000,000\, \times \,C\, \times \,{1}000} \right)/ \, \left( {N\, \times \,L} \right),$$

where FPKM (*A*) is the transcript quantification of gene *A*, *C* is the number of reads mapped to gene *A*, *N* is the total number of reads mapped to all genes, and *L* is the number of bases in gene *A*. The DESeq2 method was used to screen differentially expressed genes (DEGs) between UV-A-exposed groups and the control (UV1h vs. CK, UV2h vs. CK, UV2h vs. UV1h) using the following criteria as default: fold-change (FC) > 1.0 (log2 ratio > 1.0) and *P* < 0.05^39^.

### Quantitative real‐time RT‐PCR analysis

To further evaluate DEGs identified, we randomly selected 15 genes to quantify their expression levels by quantitative reverse transcription PCR (RT-qPCR) after UV-A exposure. RT-qPCR analysis was performed for samples previously used in RNA-seq analysis. Candidate DEG expression analysis for each of the three biological replicates per sample was performed using three technical replicates. Specific primer pairs for each gene were designed using Primer Premier 6 (Table S1). Tm (°C) is 55–60 °C, GC (%) is 50%–62% of primer, and amplification efficiency is 90.5%–100.3%. Total RNA was extracted using TRIzol reagent (Invitrogen) from different UV treatments of 3-day-old *O. furnacalis* females. The RNA obtained was reverse-transcribed in a 20 μL reaction system using the PrimeScript RT reagent Kit with genomic DNA (gDNA) Eraser (TaKaRa, Japan). Next, RT-qPCR was performed using the Applied Bio System 7500 Real-Time PCR System (Applied Biosystems, Foster City, CA, USA) with SYBR Green (Bio-Rad, Japan). Each reaction was conducted in a 20 μL mixture containing 10 μL of 2 × Universal SYBR Green Supermix, 1 μL of cDNA, 1 μL of forward primers (10 μM), 1 μL of reverse primers (10 μM), and 7 μL of diethyl pyrocarbonate (DEPC) H_2_O. The qPCR cycling parameters were as follows: 95 °C for 3 min, followed by 40 cycles of 95 °C for 30 s and 60 °C for 30 s, and melting curve generation from 65 to 95 °C. The comparative 2^−ΔΔCT^ method was used to calculate the relative quantification^40^. *β-actin* (GenBank accession no. KT366041.1) and *glyceraldehyde 3-phosphate dehydrogenase* (*GAPDH*) (GenBank accession no. KC966942.1) were used as internal reference genes for RT-qPCR analysis^83,84^.

## Metabolomics analysis

### Metabolite extraction

For metabolite extraction, 100 mg of tissue was ground with liquid nitrogen, and the homogenate was resuspended with prechilled 80% methanol and 0.1% formic acid by well-vortexing as previously described^71^. The samples were incubated on ice for 5 min and then centrifuged at 15,000 rpm for 5 min at 4 °C. Some of the supernatant was diluted to a final concentration containing 60% methanol by LC–MS-grade water. Subsequently, the samples were transferred to a new Ep tube with a 0.22 μm filter and then centrifuged at 15,000 × *g* for 10 min at 4 °C. Finally, the filtrate was injected into the LC–MS system for further analysis.

### LC–MS analysis

Metabolomic analysis was carried out as previously described^72^. Briefly, LC–MS analysis was performed using a Vanquish ultrahigh-performance liquid chromatography system (Thermo Fisher Scientific, Waltham, MA, USA) coupled with an Orbitrap Q Exactive mass series spectrometer (Thermo Fisher Scientific). The samples were injected into a Hyperil Gold column (100 × 2.1 mm, 1.9 μm) using a 16 min linear gradient at a flow rate of 0.2 mL/min. Eluents for positive (POS) ion mode were eluent A (0.1% FA in water) and eluent B (methanol), while eluents for negative (NEG) ion mode were eluent A (5 mM ammonium acetate, pH 9.0) and eluent B (methanol). The solvent gradient was set as follows: 2% B, 1.5 min; 2–100% B, 12.0 min; 100% B, 14.0 min; 100–2% B, 14.1 min; and 2% B, 16 min. The Orbitrap Q Exactive mass series spectrometer was operated in POS/NEG ion mode under the following conditions: spray voltage, 3.2 kV; capillary temperature, 320 °C; sheath gas flow rate, 35 arb; aux gas flow rate, 10 arb.

### Metabolomics data pretreatment and statistical analysis

Raw metabolite data were imported into the CD 3.1 software (Thermo Fisher Scientific), spectrum processing was performed and the database searched, quality control (QC) was performed to ensure the accuracy and reliability of the data result, and multivariate statistical analysis of metabolites was performed. Principal component analysis (PCA) and partial least squares–discriminant analysis (PLS-DA) were conducted to find differences in the metabolic composition of different alignment groups. The score plots of PLS-DA models obtained from UV-A treatment and control group pairs (UV1h–control, UV2h–control, UV2h–UV1h) were roughly separated (Fig. S3). The quality of the models was primarily instructed by the correlation coefficient (*R*^2^) of the parameter and the cross-validated correlation coefficient (*Q*^2^); the closer *R*^2^ and *Q*^2^ are to 1, the more stable and reliable the model is. Functional analysis, such as metabolic pathways, was performed to determine the biological significance of metabolites.

To identify differential metabolites (DEMs), analysis of variance (ANOVA) + *t*-test and variable influence on projection (VIP) values were used to determine statistical significance. DEMs were considered significant when *P* < 0.05 and VIP > 1.

## Results

### Transcriptomic analysis after UV-A exposure

#### *O. furnacalis* transcriptome assembly

Illumina paired-end sequencing generated 171,268,468 raw reads. After filtering, 167,314,914 clean reads were obtained, and the CK, UV1h, and UV2h cDNA libraries generated 549,285,40, 589,299,16, and 534,564,58 clean reads, respectively (Table 1). As shown in Table S2, 414,05 unigenes in the range of 201–2000 bp were assembled from clean reads with N50 lengths of 2299 bp. After eliminating repeated and short-length sequences, 50,106 unigenes were annotated against 7 public databases using similarity searching (Table S3), resulting in 20,888 unigenes (41.68%) matched to the Nt database and 19,207 unigenes (38.33%) matched to the Nr database. In total, 31,279 unigenes (62.42%) were successfully matched to at least one of the Nt, Nr, Swiss-Prot, GO, KEGG, or KOG databases, and 3,484 unigenes (6.95%) were matched to all six databases.

**Table 1:** Summary of sequences analysis for transcriptomes of *Ostrinia furnacalis* after UV-A exposure.

Sample	Raw reads	Clean reads	Clean bases	Q20 (%)	Q30 (%)	GC (%)
C	56,329,946	54,928,540	8.24G	97.63%	93.39%	45.92%
UV1h	59,849,046	58,929,916	8.84G	97.62%	93.35%	46.64%
UV2h	55,089,476	53,456,458	8.02G	97.28%	92.66%	46.75%
Total	171,268,468	167,314,914	25.1 G			

### Functional annotation of the *O. furnacalis* transcriptome

The similarity distribution of the unigenes identified showed that > 70% unigenes shared > 60% similarity with their nearest homologues (Fig. S1A). In addition, the *e*-value distribution of the unigenes identified showed that 33% unigenes shared the highest homology with an *e*-value cut-off < 1e^−100^ (Fig. S1B). As expected, the highest hits were from insect genomes, with Lepidoptera *Amyelois transitella* (15.8%), *Spodoptera litura* (11.4%), *H. armigera* (11%), and *Heliothis virescens* (8.8%) accounting for the top four unigenes with Nr annotations (Fig. S1C).

**Figure 1:**
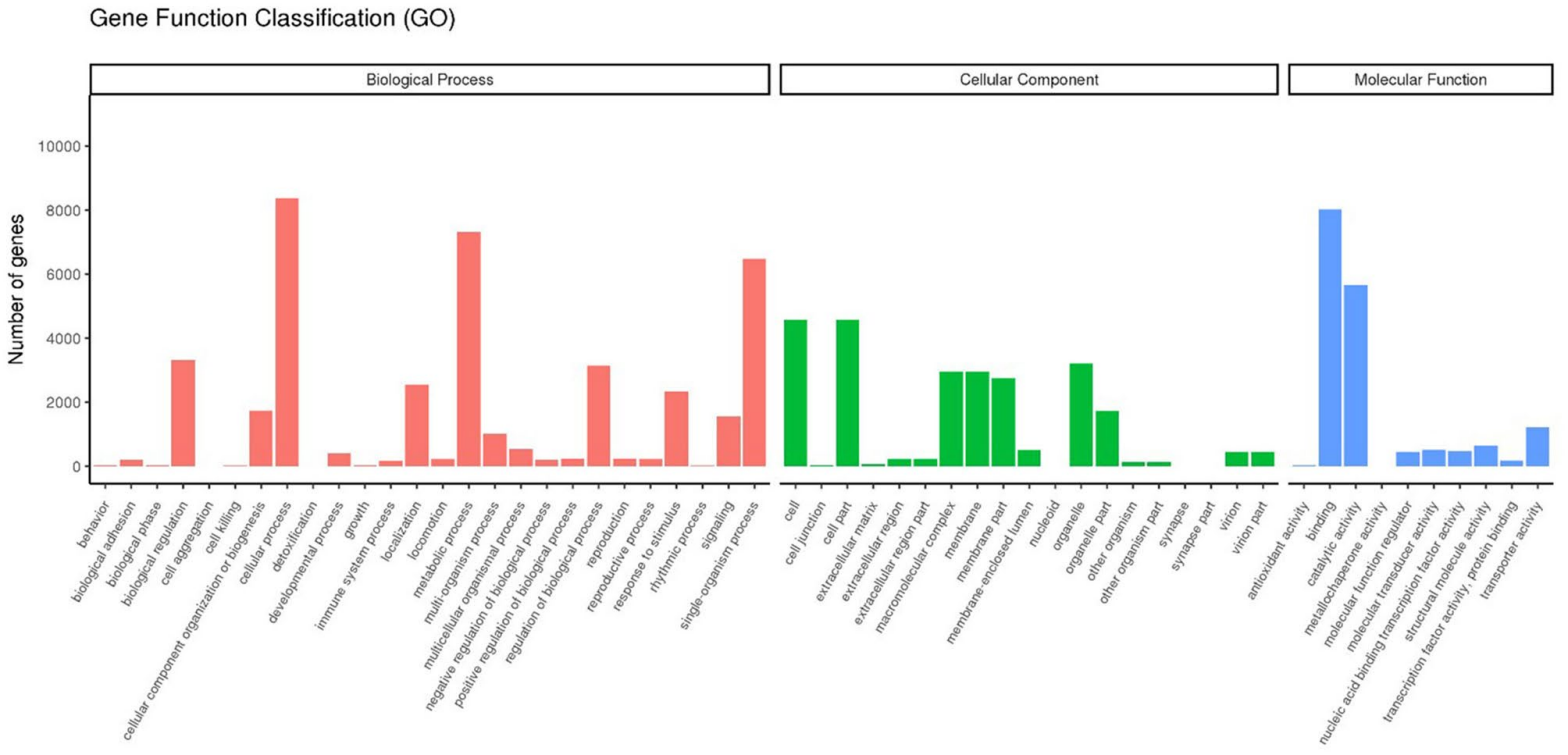
GO classification. The genes were summarized in three main categories: biological process (pink), cellular component (green), and molecular function (blue). The *y* axis represents the number of genes in the main category. *GO* gene ontology.

GO, KOG, and KEGG analyses predicted 28,812 unigenes from the functional annotation of *O. furnacalis* unigenes. The GO gene functional classification system divided 14,563 Nr unigenes into three major functional ontologies: biological process, with 26 subcategories; cellular component, with 19 subcategories; and molecular function, with 10 subcategories (Fig. 1). The top 10 subcategories were cellular process (8363), binding (8015), metabolic process (7311), single-organism process (6480), cell (4570), cell part (4570), catalytic activity (5660), biological regulation (3316), organelle (3201), and biological process regulation (3135). In GO analysis, developmental process (392), localization (2551), locomotion (212), reproduction (232), reproductive process (215), response to stimulus (2337), and rhythmic process (10) were categorized under “biological process.”

**Figure 2:**
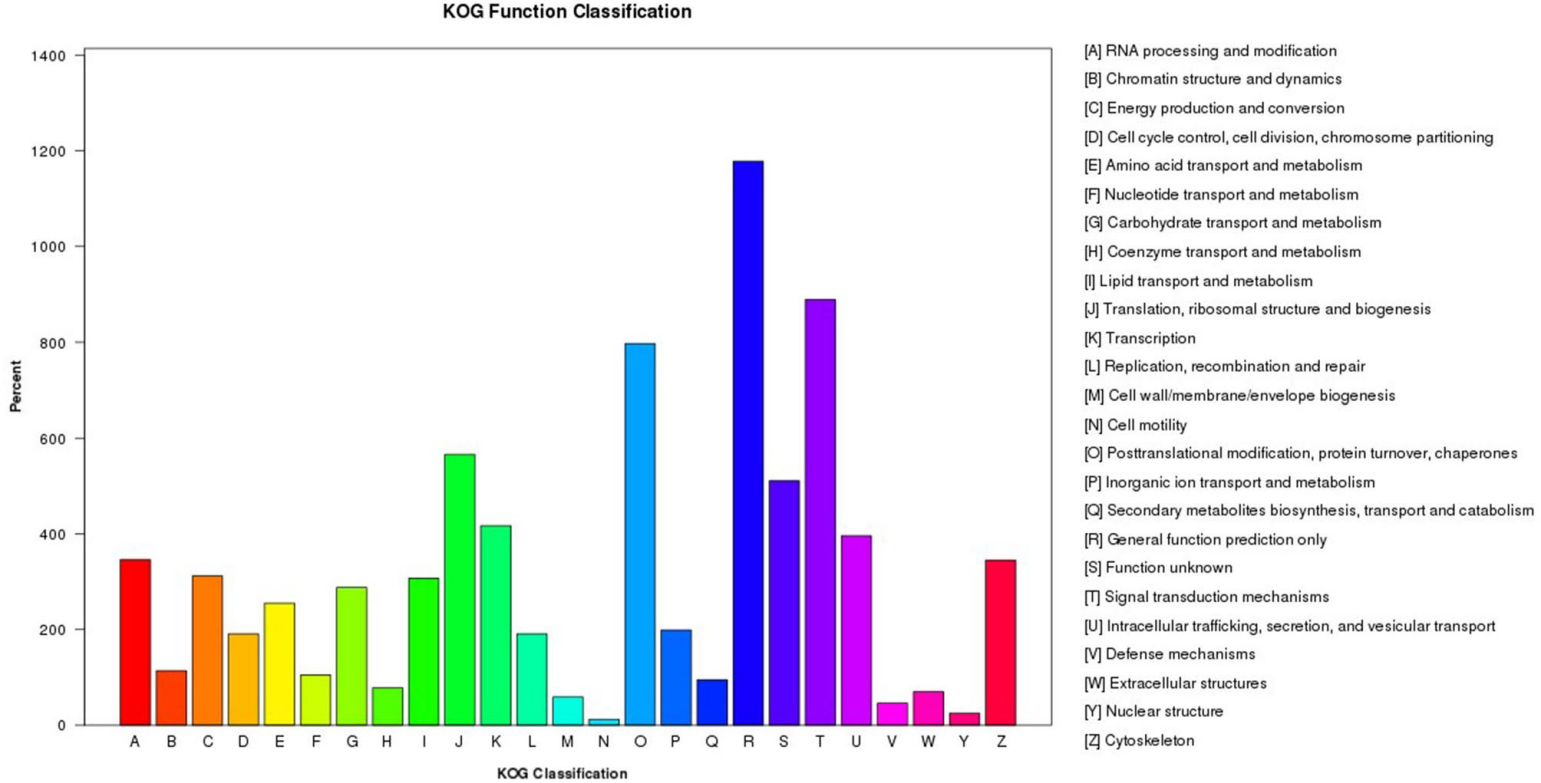
Distribution of All-unigenes according to the KOG functional classification. *KOG* EuKaryotic Orthologous Groups.

The KOG functional classification divided the 7028 assembled All-unigenes into 26 categories (Fig. 2), of which the top 5 were translation and general function prediction only (16.76%); signal transduction mechanisms (12.65%); posttranslational modification, protein turnover, and chaperones (11.34%); translation, ribosomal structure, and biogenesis (8.05%); and transcription (5.93%). In contrast, the least represented categories were nuclear structure (0.36%) and cell motility (0.17%).

**Figure 3:**
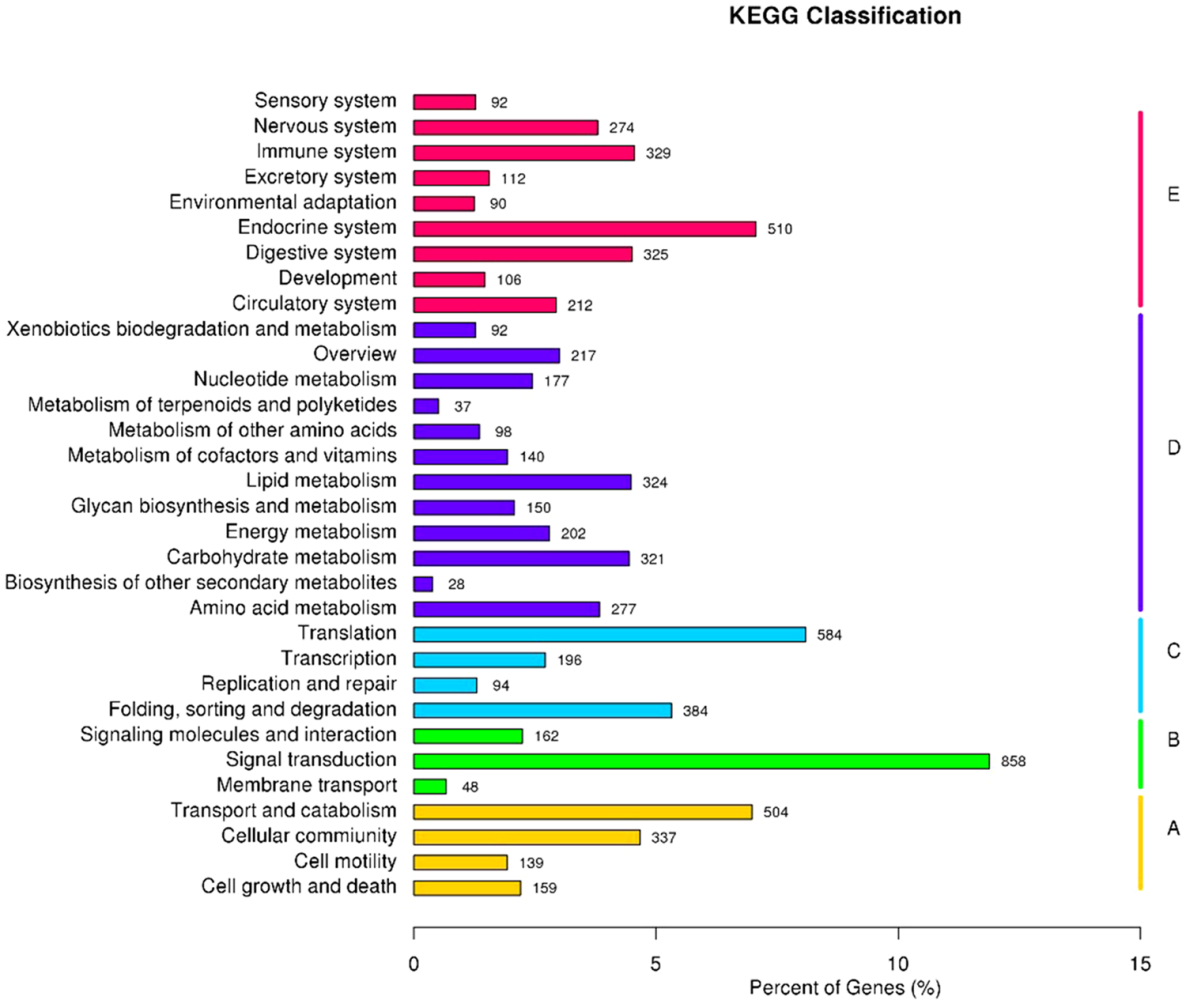
Mapping the KEGG pathway of *Ostrinia furnacalis*. **(A)** Cellular processes; **(B)** environmental information processing; **(C)** genetic information processing; **(D)** metabolism; (**E**) organismal systems. *KEGG* Kyoto Encyclopedia of Genes and Genomes.

The 7,221 assembled All-unigenes were assigned to 228 KEGG pathways belonging to 5 KEGG categories (Fig. 3): cellular processes, environmental information processing, genetic information processing, metabolism, and organismal systems. Of these, signal transduction (858 unigenes; 11.88%), translation (584 unigenes; 8.0,%) and endocrine system (510 unigenes; 7.1%) contained the largest number of unigenes.

### *O. furnacalis* genes differentially expressed in UV-A exposure response

**Figure 4:**
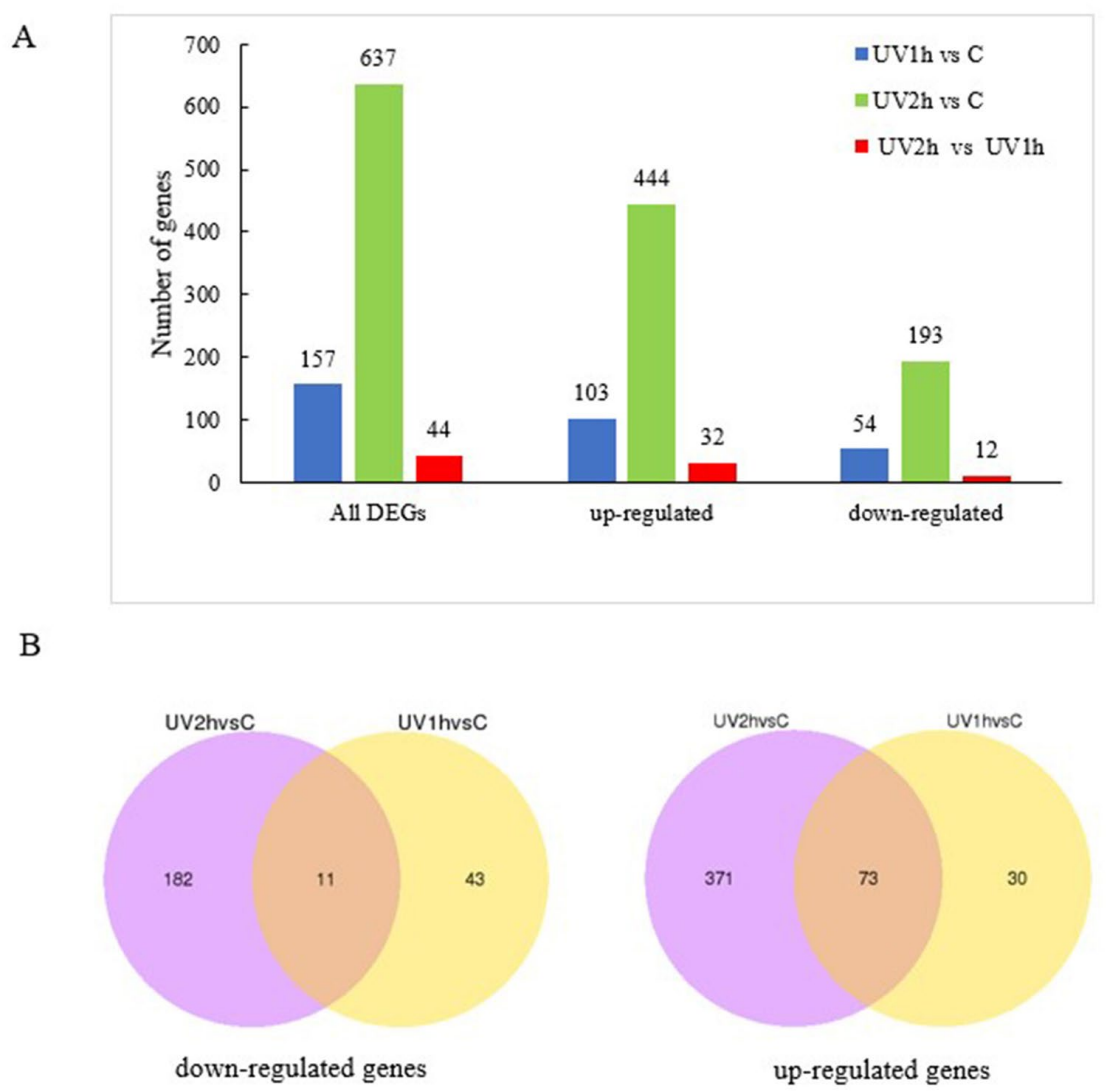
DEGs in *Ostrinia furnacalis* after UV-A exposure. **(A)** Histogram of the number of annotated unigenes in *O. furnacalis* after UV-A exposure. **(B)** Venn diagrams of common differential expression of upregulated (right) and downregulated genes (left) between UV-treated and control groups. *DEGs* differentially expressed genes, *UV* ultraviolet.

After UV-A exposure, 157 and 637 DEGs were detected in UV1h and UV2h, respectively, compared to CK (no UV-A exposure), with 103 and 444 genes, respectively, being upregulated and 54 and 193 genes, respectively, being downregulated. In addition, 44 DEGs were detected in UV2h compared to UV1h (Fig. 4A). The expression of most of these DEGs increased, and 84 DEGs (73 upregulated, 11 downregulated) showed common differential expression in *O. furnacalis* after UV-A exposure (Fig. 4B).

Of the three GO categories (biological process, cellular component, and molecular function) to which DEGs were assigned (Fig. S2), “biological process” formed the largest group, followed by “cellular component” and “molecular function.” In “biological process,” single-organism process, cellular process, and metabolic process subcategories were dominant in the UV-A exposure response. In “cellular component,” most of the DEGs were enriched in membrane, cell, and cell part subcategories, while in “molecular function,” most of the DEGs were enriched in binding and catalytic activity subcategories.

KEGG database mapping showed that most of the DEGs were related to ABC transporters, followed by amyotrophic lateral sclerosis and phototransduction-fly in UV1h compared to CK. In addition, most of the DEGs were related to focal adhesion, bile secretion, and ABC transporters in UV2h compared to CK (Tables S3–S5). On the basis of Nr and Pfam annotation, many DEGs were implicated in signal transduction, immune response, antioxidant system and detoxification, and stress response genes, such as lipoprotein-related protein 2 (*LRP2*), catalase (*CAT*), calmodulin (*CALM*), choline dehydrogenase (*CHDH*), serine/threonine-protein kinase (*CST*), chitinase 3 (*Chit3*), *p450s*, aldehyde dehydrogenase (*ALDH*), c-Jun N-terminal kinase (*JNK*), and Ras guanosine triphosphatase *(GTPase)-*activating protein (*RasGAP*). Of the DEGs detected between UV1h and UV2h, genes involved in immune response and signal transduction, including chitin binding-like protein (*CBP*) and ATP-binding cassette subfamily A member 3 (*ABCA3*), were overrepresented after UV-A exposure (Table 2), indicating that they might increase adaptation of *O. furnacalis* to UV-A stress.

**Table 2:** List of DEGs in *O. furnacalis* under UV-A exposure.

Genes	UV1h vs. CK	UV2h vs. CK	UV2h vs. UV1h
**Immune system**			
Serine/threonine-protein kinase (CST)	UP	UP	N/A
Chitinase 3 (Chit3)	N/A	UP	N/A
Kallikrein 1 (KLKB1)	N/A	UP	N/A
G protein-coupled receptor (GPCR)	N/A	UP	N/A
Chitin binding-like protein (CBP)	N/A	UP	UP
**Signal transduction**			
Lipoprotein-related protein 2 (LRP2)	UP	UP	N/A
ATP-binding cassette subfamily A member 3 (ABCA3)	N/A	N/A	UP
Catalase (CAT)	UP	UP	N/A
Calmodulin (CALM)	UP	UP	N/A
Collagen, type IV, alpha (COL4A)	N/A	UP	N/A
Hypoxia-inducible factor 1 alpha (HIF1A) GTP-binding protein	N/A N/A	UP UP	N/A N/A
**Antioxidant system**			
Catalase (CAT)	UP	UP	N/A
Peroxidase (POD)	N/A	UP	N/A
**Detoxification and stress‐response genes**			
Cytochrome P450 monoxygenases (CYP450s)	UP	UP	N/A
UDP-glucuronosyltransferases (UGTs)	N/A	UP	N/A
Aldehyde dehydrogenase (ALDH) Carboxylesterase (CarE) c-Jun N-terminal kinase (JNK) Ras GTPase-activating protein (RasGAP)	N/A N/A UP N/A	UP UP UP UP	N/A N/A N/A N/A

*N/A* not significantly changed, *DEGs* differentially expressed genes, *UV* ultraviolet.

### RT-qPCR verification of transcriptomic data

**Figure 5:**
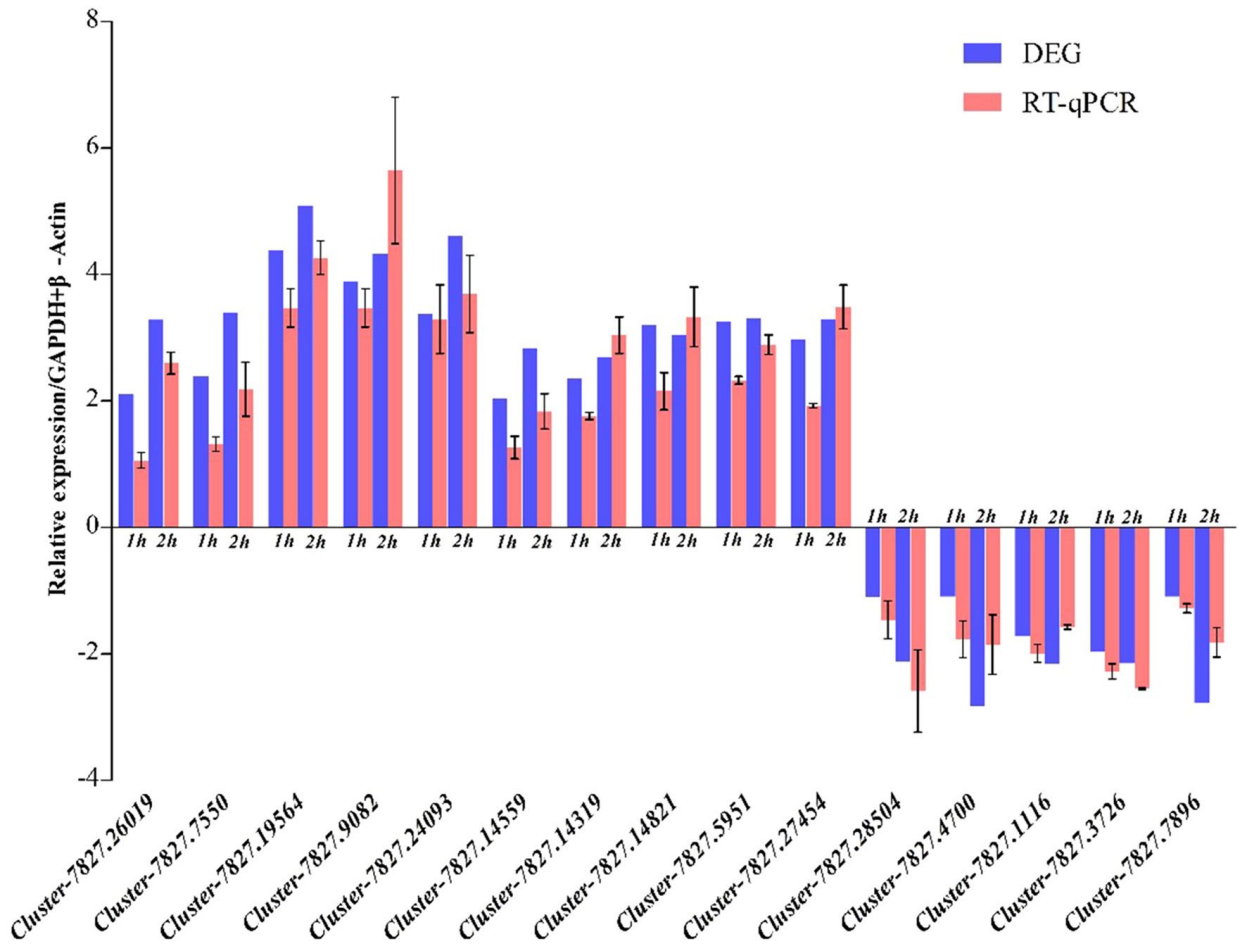
qRT-PCR validation of 15 selected DEGs. *GAPDH* and *β-actin* were used as internal controls. The *x* axis represents DEGs, whereas the *y* axis represents the relative expression levels of genes. *qRT-PCR *quantitative reverse transcription polymerase chain reaction, *DEGs* differentially expressed genes, *GAPDH* glyceraldehyde 3-phosphate dehydrogenase.

Thousands of genes from transcriptome libraries showed significantly different expression levels. Most of the 15 DEGs (TBC1 domain family member 22B, facilitated trehalose transporter Tret1-like; protein yellow-like; glucose dehydrogenase [FAD, quinone]-like; excitatory amino acid transporter-like; UNC93-like protein; sugar transporter ERD6-like; transcript variant X3; catalase-like; orexin receptor type1-like; TGF-beta-activated kinase 1 and MAP3K7-binding protein 1-like; down syndrome cell adhesion molecule-like protein; SET and MYND domain-containing protein 4; sodium/potassium-transporting ATPase subunit beta-2-like; lutropin-choriogonadotropic hormone receptor-like) selected had expression patterns similar to transcriptome data (Fig. 5). The expression patterns of all 15 DEGs matched sequencing data. Therefore, these comparative analyses validated the accuracy and reliability of RNA-seq data.

### Metabolomic analysis after UV-A exposure

#### Multivariate statistical analysis

**Figure 6:**
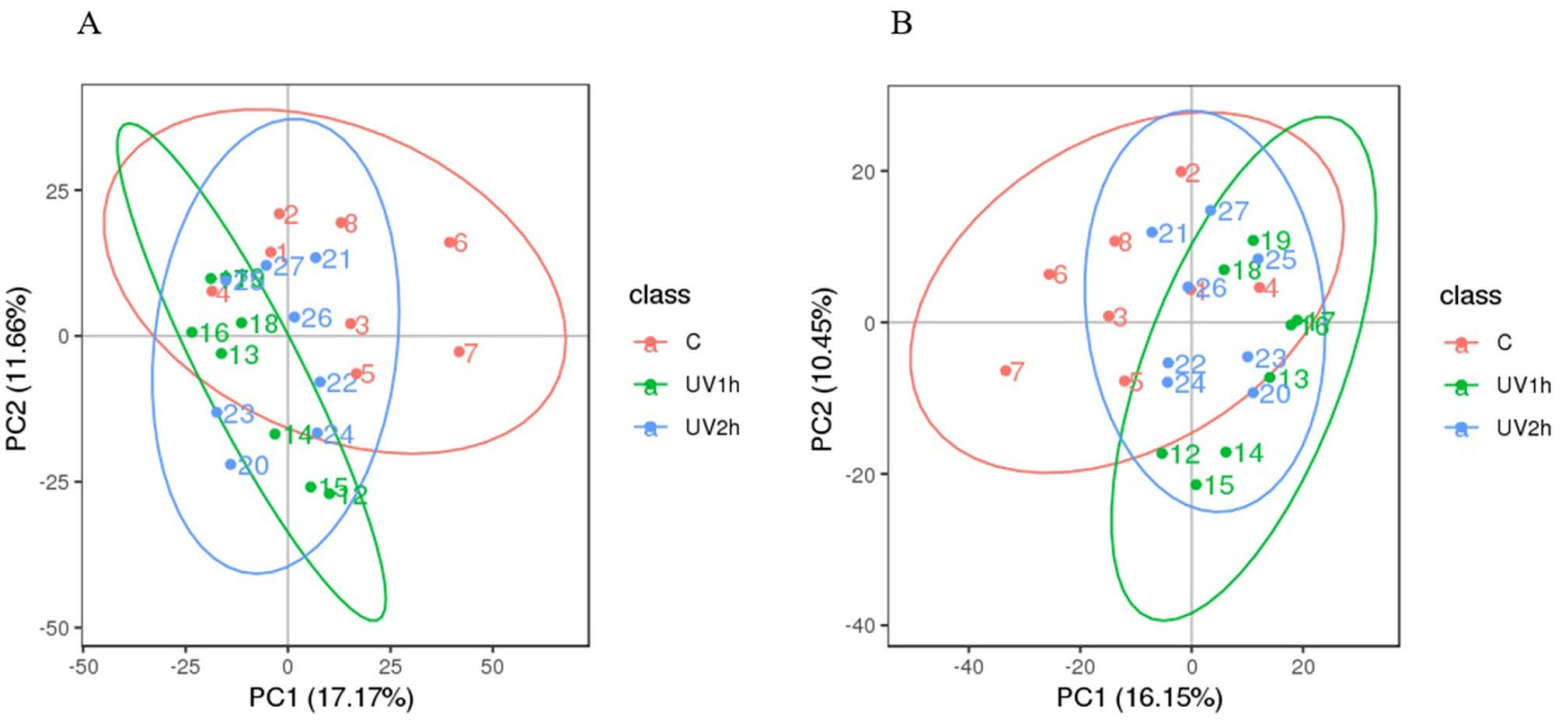
PCA of metabolic profiles of CK, UV1h, and UV2h (eight biological replicates) identified in POS **(A)** and NEG **(B)** ion modes. *PCA* principal component analysis, *UV* ultraviolet, *POS* positive, *NEG* negative, *PC1* first principal component, *PC2* second principal component.

After QC, we detected 1756 and 1088 peaks using LC–MS in POS and NEG ion modes, respectively. After Pareto scaling treatment, the PCA diagram constructed for multivariate variable pattern recognition analysis revealed a clear separation in metabolite accumulation in UV1h, UV2h, and CK in both POS and NEG ion modes (POS: PC1, 17.17%; PC2, 11.66%; NEG: PC1, 16.15%; PC2, 10.45%) (Fig. 6A,B), indicating significant changes in metabolites between CK and UV1h and UV2h. However, there was no significant difference in metabolites between UV2h and UV1h.

### Differential metabolite analysis

The *R*^2^ values of the six models were > 0.89 (Table 3). Permutation tests (*n* = 200; Fig. S4) showed that all PLS-DA models were robust with no overfitting. Filtering (VIP > 1 and *P* < 0.05 [*t*-test]) acquired DEMs for the separation of different groups. In POS ion mode, we screened 181 (up 42, down 139) (Fig. S5A), 111 (up 46, down 65) (Fig. S5B), and 34 (up 28, down 6) (Fig. S5C) DEMs from the PLS-DA models. In NEG ion mode, we screened 135 (up 32, down 103) (Fig. S5D), 93 (up 38, down 55) (Fig. S5E), and 36 (up 31, down 5) (Fig. S5F) DEMs from the PLS-DA models for intergroup comparative analysis.

**Table 3:** Parameters of projections to PLS-DA models on the basis of different comparative analyses.

Ion detection mode	Comparative analysis	*R*^2^	*Q*^2^
Positive (POS)	UV1h vs. C	0.91	0.59
	UV2h vs. C	0.92	0.48
	UV2h vs. UV1h	0.94	0.44
Negative (NEG)	UV1h vs. C	0.89	0.63
	UV2h vs. C	0.91	0.50
	UV2h vs. UV1h	0.93	0.51

*PLS-DA* partial least squares-discriminant analysis, *UV* ultraviolet.

By comparing the number of DEMs in *O. furnacalis* between UV1h, UV2h, and CK, we detected 316 DEMs in UV1h and 204 DEMs in UV2h with the same UV-A dose compared to CK. However, only 70 DEMs were detected in UV2h compared to UV1h.

A part of saccharides, amino acids, fatty acids, and organic acids significantly changed after UV-A exposure. The expression levels of some metabolites, such as phosphoarginine, L-aspartic acid, L-serine, arachidonic acid, ethyl linoleate, and phenylglyoxylic acid, significantly increased in UV1h compared to CK. In contrast, other metabolites, such as glucose, *N*-acetylornithine, d-tryptophan, and azelaic acid, were downregulated. Similarly, the expression levels of some metabolites, such as d-glucopyranoside, *N*-acetylleucylleucine, arachidonic acid, adipic acid, benzoic acid, and phenylglyoxylic acid, significantly increased in UV2h compared to CK. In contrast, other metabolites, such as glucose, acetyl-l-methionine, l-alanine, and azelaic acid, were downregulated. In addition, the expression levels of most metabolites, such as 3-phosphoglyceric acid, tryptoline, thyronine, gamma-l-glutamyl-l-tyrosine, d-α-hydroxyglutaric acid, and phthalic acid, significantly increased in UV2h compared to UV1h. Details of these differences are given in Tables S6–S8.

### KEGG pathway analysis of different metabolites

The DEM pathways in UV1h, UV2h, and CK were biosynthesis of amino acids, histidine metabolism, nicotinate and nicotinamide metabolism, arachidonic acid metabolism, citrate acid cycle (tricarboxylic acid [TCA] cycle), and pantothenate and coenzyme A (CoA) biosynthesis (Tables S9–S10).

### Transcriptome and metabolome association analysis using KEGG

**Figure 7:**
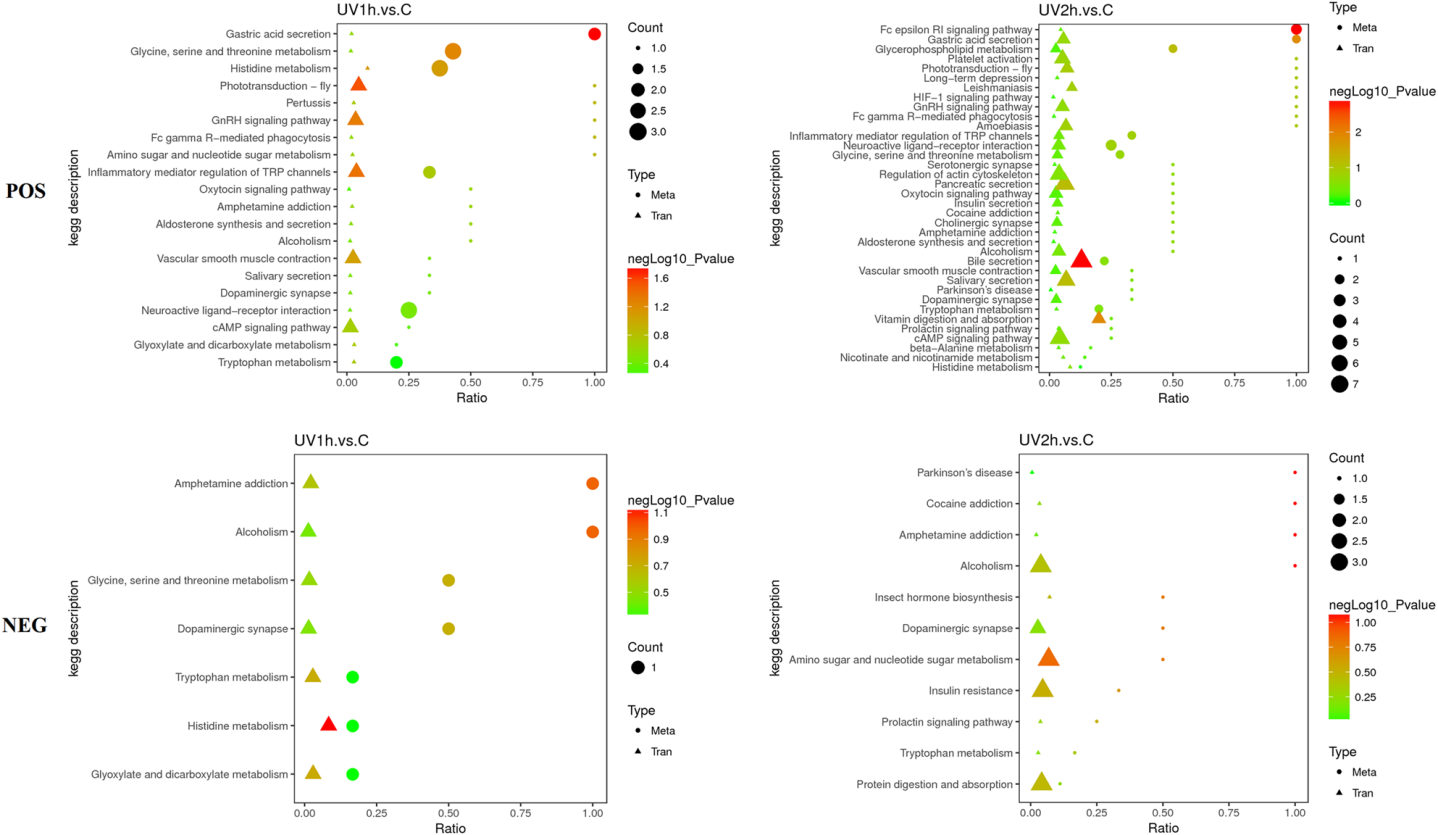
Association analysis of DEG and DEM KEGG pathway. *POS* positive, *NEG* negative, *Count* number of genes or metabolites enriched in the pathway, *Type* “Tran” indicates transcriptome, while “Meta” means metabolome, *DEG* differentially expressed gene, *DEM* differently expressed metabolites, *KEGG* Kyoto Encyclopedia of Genes and Genomes.

Association analysis revealed that in POS ion mode, 20 and 36 pathways were conjointly involved in DEGs and DEMs in UV1h and UV2h, respectively, compared to CK. Similarly, in NEG ion mode, 7 and 10 pathways were conjointly involved in DEGs and DEMs in UV1h and UV2h, respectively, compared to CK (Fig. 7). The main KEGG pathways, including protein digestion and absorption, amino sugar and nucleotide sugar metabolism, insect hormone biosynthesis, and tryptophan metabolism, are shown in Fig. 7. There are common DEGs and DEMs in the cAMP signaling pathway, amino sugar and nucleotide sugar metabolism, dopaminergic synapse, gonadotropin-releasing hormone (GnRH) signaling pathway through transcriptome, and metabolome association analysis in *O. furnacalis* under UV-A exposure (Table 4).

**Table 4:** List of common DEGs and DEMs in *O. furnacalis* under UV-A exposure.

Comparative analysis	KEGG pathway	Type	P value	Count	Meta/Tran name	Up/down
UV1h vs C	cAMP signaling pathway	DEM	0.454	1	Acetylcholine	Up
		DEGs	0.214	2	Hypothetical protein B5V51_8840, partial calcium-binding protein E63-1 isoform X1	Up Up
	Amino sugar and nucleotide sugar metabolism	DEM	0.139	1	UDP-*N*-acetyl-3-*O*-(1-carboxyvinyl)-alpha-d-glucosamine	Down
		DEG	0.236	1	chitinase 2	Up
	Dopaminergic synapse	DEM	0.199	1	l-Dopa	Down
		DEG	0.359	1	Calcium-binding protein E63-1 isoform X1	Up
	GnRH signaling pathway	DEM	0.139	1	Arachidonic acid	Up
		DEGs	0.048	2	Serine..threonine-protein kinase CST20 isoform X1 Calcium-binding protein E63-1 isoform X1	Up Up
UV2h vs C	cAMP signaling pathway	DEM	0.401	1	Arachidonic acid	Up
		DEGs	0.234	6	Sodium..potassium-transporting ATPase subunit beta-2-like calcium-binding protein E63-1 isoform X1 Hypothetical protein B5V51_8840, partial dopamine D2-like receptor Multidrug resistance-associated protein 4-like isoform X1 Sodium..potassium-transporting ATPase subunit beta-2-like	Up Up Up Down Up Down
	Amino sugar and nucleotide sugar metabolism	DEM	0.165	1	UDP-*N*-acetylglucosamine	Up
		DEGs	0.142	3	Cytochrome P450 4C1-like chitinase 2 Chitinase-3-like protein 2	Up Up Up
	Dopaminergic synapse	DEM	0.364	1	l-Dopa	Down
		DEG	0.359	1	Calcium-binding protein E63-1 isoform X1	Up
	GnRH signaling pathway	DEM	0.119	1	Arachidonic acid	Up
		DEGs	0.233	3	Serine..threonine-protein kinase CST20 isoform X1 Ras, partial calcium-binding protein E63-1 isoform X1	Up Up Up

## Discussion

Environmental changes pose a great threat to the survival and reproduction of insects, and insects must have a certain tolerance ability to adapt to these changes^75,76^. Insects sense the external environment stimulation and transmit the signal to the body to respond to environmental stresses^43^. Most insects have developed various mechanisms, such as morphological and physiological adaptations, to cope with environmental stresses, such as high temperature, cold stress, UV radiation, and bacterial and fungal infections^32,77–79^. Solar UV radiation is one of the most omnipresent environmental stresses^41,42^. In addition, UV-A augmentation decreases adult longevity, increases the oviposition rate, and prolongs developmental periods of the F_1_ generation^,^^31^. A comparative proteomic study demonstrated that differentially expressed proteins of *O. furnacalis* under UV-A stress are involved in diverse biological processes, such as signal transduction, cellular stress response, transport processing, cytoskeleton organization, and metabolism^43^.

The response patterns of insects are different under different periods of UV-A stress. A previous study showed that *H. armigera* adults exposed to UV light at certain time points (from 30 to 90 min) show significantly higher total antioxidant capacity compared to the control^80^. Ali et al*.* (2017) showed that the UV-A stress response of *Mythimna separata* adults is significantly different under relatively short exposure (60 min) compared to long exposure (120 min) ^12^. One study indicated that the expression level of JNK gene in *H. armigera* significantly increases after 60 min UV-A exposure and significantly decreases after 120 min^14^. These findings were consistent with our results, indicating that more and more genes are involved in the UV-A stress response of *O. furnacalis* as time goes on. Therefore, the selection of UV-A radiation time is important to study the molecular mechanism underlying the UV-A stress response in *O. furnacalis* and to screen functional genes. GO enrichment items of DEGs are almost the same after 1 and 2 h UV-A exposure, and most are related to metabolic process, cellular process, single organization process, cell, cell part, catalytic activity, and binding functions. This result is similar to the GO enrichment result of *Glyphodes pyloalis* DEGs under heat stress^22^.

Insects transmit external environmental stimuli to the cellular machinery through signal transduction pathways. Signal transduction pathway–associated genes, such as *LRP2*, *ABCA3*, *CALM*, *COL4A*, *HIF1A*, and *GBP*, significantly change. As molecular switches, they frequently participate in pathways regulating many cellular processes of cell growth, differentiation, and morphogenesis^44^. In insects, the innate immune system is the primary effector response system^45^. Remarkably, a series of immune-related genes, such as *CST*, *Chit3*, kallikrein 1 (*KLKB1*), G protein-coupled receptor (*GPCR*), and *CBP*, significantly change after UV-A exposure. Maladjustment of immune-related genes might destroy the immune defense system. Previous studies reported that UV exposure increase ROS activity and cause oxidative stress in *H. armigera* adults and *Myzus persicae* ^13,81^. CAT is a vital element of insects' antioxidant system; it is also a light-sensitive antioxidant enzyme. It can efficiently catalyze H_2_O_2_ decomposition to maintain equilibrium between de novo H_2_O_2_ generation and effective elimination, which is crucial for innate immunity^46^. POD is responsible for enhancing stress tolerance of living organisms^47^. Both CAT and POD are significantly upregulated after UV-A exposure, which is in agreement with results reported for *Spodoptera litura* and *Locusta migratoria tibetensis*^48,82^. Therefore, UV-A exposure might lead to oxidative stress in *O. furnacalis*.

Detoxification may be the other critical accommodative strategy in response to UV-A stress. UV-A exposure leads to the expression of various genes involved in detoxification, such as *UGTs*, *p450s*, and *ALDH*, which are significantly upregulated after UV-A exposure. *P450s* and *UGTs* separately act as phase I and phase II metabolic enzymes and play an extremely important role in the disinteration of endobiotics and xenobiotics^49^. Sang et al*.* (2012) reported that UV-A exposure can affect *P450* expression in *Tribolium castaneum*. The upregulated expression levels of *P450* and *UGT* constitute a powerful detoxification metabolic system in *O. furnacalis* in response to UV-A stress. Through catalyzing their oxidation to nonreactive acids, ALDHs achieve elimination of toxic aldehydes, the enzyme activity of which is primarily related to the metabolism of endogenous lipid peroxidation products^50^. The ALDH mRNA is significantly upregulated after UV-A exposure, indicating that it contributes to improved UV-A stress tolerance via the metabolic pathway. As an important detoxification enzyme family, CarE participates in the detoxification and metabolism of many drugs, carcinogens, and environmental toxins^51^. Zhao et al*.* (2017) reported that CarE activity in *Plutella xylostella* does not significantly change in low-concentration Cry1Ac toxin treatment groups^52^, while CarE activity in high-concentration Cry1Ac toxin treatment groups is higher compared to the control. In addition, when *H. armigera* was exposed to UV radiation for 30, 60, and 90 min, CarE activity in the body was significantly lower compared to the control^6^. Our finding that CarE is significantly upregulated after UV-A exposure for 2 h maybe indicates that *O. furnacalis* can adapt for a longer period to UV-A stress*.* These results suggest that p450s, UGTs, ALDHs, and CarE might assist in adaptation and/or UV-A resistance.

As reported in earlier studies on *Drosophila melanogaster*^16^, MAPK pathways are associated with UV-A stress. MAPKs control a plethora of physiological processes^53^. *RasGAP* and *JNK* are upstream and downstream genes, respectively, in the MAPK pathway^54,55^. JNK activation is also triggered by Ras activation^56^. Ruan et al*.* (2015) reported that *RasGAP* expression can be induced in *Microdera punctipennis* by environmental stress^57^. *JNK* mRNA expression in *H. armigera* adult females is induced by UV-A stress and reaches the highest expression level at 60 min postexposure^14^. Our previous study reported that *JNK* in *O. furnacalis* shows increased expression after UV-A exposure for 60 min. *RasGAP* and *JNK* are upregulated in *O. furnacalis* under UV-A stress, indicating that MAPK pathways in *O. furnacalis* might involve UV-A exposure–induced stress responses^32^.

Short-term UV-A exposure induces metabolic perturbation in *O. furnacalis*; with an increase in radiation time, the adaptability of *O. furnacalis* to UV-A radiation gradually increases, and the metabolites gradually return to a stable condition. However, UV-A exposure for 1 and 2 h has little significant difference. In addition, nearly half of the metabolite content is downregulated in *O. furnacalis* after UV-A exposure, which may be because of UV-A stress. Protecting from damage induced by UV-A stress and continuously enhancing resistance and defense definitely consumes a lot of energy, leading to a decrease in metabolite levels^31,58^.

Most amino acids, such as aspartic acid, tyrosine, alanine, methionine, serine, tryptophan, arginine, and citrulline are significantly changed in *O. furnacalis* after UV-A exposure. Alanine, aspartic acid, and tyrosine are crucial intermediates in the TCA cycle as precursors of acetyl-CoA, oxaloacetic acid, and α-ketoglutarate^59,60^. Tryptophan and tyrosine act as antioxidants because they demonstrate rapid, non‐enzymatic repair by a tocopherol analog, ascorbate, and trolox, as well as methionine. Methionine also acts as an antioxidant to protect cells from UV-A stress damage^61^. In addition, some metabolites, such as arginine and citrulline, play an important role in regulating gene expression^62,63^. These results indicate that the perturbation of amino acid metabolism in *O. furnacalis* might be caused by UV-A exposure.

UV-A exposure downregulates several metabolites, such as glucose, d-glucopyranoside, d-xylopyranoside, d-erythro-pentopyranose, glucuronamide, and *N*-acetyl-d-glucosaminitol, perhaps as a response to increased energy exertion under UV-A exposure. Citric acid as a key metabolite in the TCA cycle is upregulated after UV-A exposure, indicating that UV-A exposure affects the entire energy metabolism of *O. furnacalis* adult females^92^. The increase in energy metabolism leads to an increase in the demand for oxygen in insects, which, in turn, leads to ROS formation in aerobic respiration in insects^64^, and ROS can cause DNA damage^65^, thus increasing the burden on insects to repair UV-A exposure induced DNA damage. This result agrees with findings of Meng et al*.* (2009), who reported that solar UV radiation increases oxidative stress, affects antioxidant enzyme activity, and disturbs insect physiology^13^.

Lipids have vital functions in energy storage in organisms and play numerous roles in structure and biological processes. In addition, fatty acids are metabolic fuels and ingredients of lipid conformation in organisms. Some free fatty acids, such as arachidonic acid, ethyl linoleate, docosahexaenoic acid, and adipic acid, are upregulated after UV-A exposure, which means that UV-A exposure significantly promotes fatty acid synthesis. Docosahexaenoic acid decreases lipid peroxide accumulation and ROS in the central nervous system and also enhances glutathione reductase, leading to antioxidative activity^66–68^. Arachidonic acid is a necessary fatty acid and is reposited in membrane phospholipids, which participate in multiple physiological functions and as a nutritional supplement^69^. In addition, UV-A exposure downregulates some fatty acids, such as tetradecanedioic acid, dodecanedioic acid, hexadecandioic acid, and azelaic acid. A decrease in these fatty acids shows that UV-A exposure disrupts part of lipid metabolism in *O. furnacalis*.

The reproductive processes of female insects have been studied extensively because of their importance for species propagation and great potential as targets for insect control^85^. JH regulates adult reproduction by regulating the synthesis and absorption of vitellogenin (Vg)^86,87^. The expression of JH III, which plays an important role in the reproduction of lepidopteran insects, was downregulated under UV-A exposure in *O. furnacalis*. This observation was consistent with the results of Lopez–Martinez et al*.* (2008) and Zhang et al*.* (2012). The expressions of Vg and Vg receptor (VgR) were upregulated in *O. furnacalis* under UV-A exposure, which is in agreement with our previous result that UV-A could induce *OfVg* and *OfVgR* expression in *O. furnacalis*^31^*.*

Comparative transcriptomic and metabolomic analysis showed that some DEGs could affect the content of DEMs in *O. furnacalis*. As a DEG, the calcium-binding protein gene was involved in the regulation of various DEMs in *O. furnacalis*, such as citric acid, acetylcholine, arachidonic acid, and levodopa (l-Dopa), in our study. We speculate that it is due to the participation of the Ca^2+^ binding protein gene in apoptosis^88,89^. A previous study has reported that uridine diphosphate (UDP)-*N*-acetylglucosamine was involved in the development, survival, and molting of insects^90^. Our results showed that the synthesis of UDP-N-acetylglucosamine was significantly increased in response to UV stress, which was mainly regulated by the expression of cytochrome P450 4C1-like, chitinase 2, chitinase-3-like, and protein 2. In addition, we found that arachidonic acid was regulated by several DEG containing calcium-binding protein E63-1 isoform X1, dopamine D2-like receptor, and multidrug resistance-associated protein 4-like isoform X1 in the cAMP signaling pathway. Lee et al*.* (2001) reported that the cAMP signaling pathway is involved in DNA damage response and repair of UV-damaged DNA lesions^91^. This is consistent with the results of our study.

## Conclusion

Comparative transcriptomic and metabolomic analyses were implemented in UV1h, UV2h, and CK. Most of the DEGs in *O. furnacalis* were involved in signal transduction, immune defense, antioxidative system, detoxification, and stress response and metabolic pathways after UV-A exposure. Metabolomics data showed that perturbation of amino acid, sugar, and lipid metabolism might be the main UV-A stress response. This study markedly extends our comprehension of adaptive mechanisms in insects under UV-A exposure and also indicates that *O. furnacalis* as a nonmodel insect has conserved response pathways under UV-A stress.

## Supplementary Information

Supplementary Information.

## References

[1] Paul N (2003). Ecological roles of solar UV radiation: Towards an integrated approach. Trends Ecol. Evol..

[2] Smedley ARD, Rimmer JS, Moore D, Toumi R, Webb AR (2012). Total ozone and surface UV trends in the United Kingdom: 1979–2008. Int. J. Climatol..

[3] Rünger TM (2008). Kappes UP Mechanisms of mutation formation with long-wave ultraviolet light (UV-A). Photodermatol. Photo..

[4] Briscoe AD, Chittka L (2001). The evolution of colour vision in insects. Annu. Rev. Entomol..

[5] Meyer-Rochow VB, Kashiwagi T, Eguchi E (2002). Selective photoreceptor damage in four species of insects induced by experimental exposures to UV-radiation. Micron.

[6] Meng JY, Zhang CY, Lei CL (2012). The effects of UV light stress on acetylcholinesterase and carboxylesterase in *Helicoverpa armigera* adults. Guizhou Agric. Sci..

[7] Zhang CY, Meng JY, Wang XP, Zhu F, Lei CL (2011). Effects of UV-A exposures on longevity and reproduction in *Helicoverpa armigera*, and on the development of its F1 generation. Insect Sci..

[8] Zhang CY, Meng JY, Zhou LJ, Sang W, Lei CL (2012). Effects of ultraviolet light stress on juvenile hormone in *Helicoverpa armigera* adults. Plant Prot..

[9] Girard PM, Francesconi S, Pozzebon M, Graindorge D, Rochette P, Drouin R, Sage E (2011). UV-A-induced damage to DNA and proteins: direct versus indirect photochemical processes. JPCS..

[10] Lopez-Martinez G, Elnitsky MA, Benoit JB, Lee RE, Denlinger DL (2008). High resistance to oxidative damage in the Antarctic midge *Belgica antarctica*, and developmentally linked expression of genes encoding superoxide dismutase, catalase and heat shock proteins. Insect Biochem. Mol. Biol..

[11] Suzuki T, Takashima T, Izawa N, Watanable M, Takeda M (2008). UV radiation elevates arylalkyla-mine N-acetyltransferase activity and melatonin content in the two-spotted spider mite, *Tetranychus urticae*. J. Insect Physiol..

[12] Ali A, Rashid MA, Huang QY, Lei CL (2017). Influence of UV-A radiation on oxidative stress and antioxidant enzymes in *Mythimna separata* (Lepidoptera: Noctuidae). Environ. Sci. Pollut. Res..

[13] Meng JY, Zhang CY, Zhu F, Wang XP, Lei CL (2009). Ultraviole light-induced oxidative stress: effects on antioxidant response of *Helicoverpa armigera* adults. J. Insect Physiol..

[14] Liu XF, Meng JY, Zhao XC, Zhang CY (2019). cDNA cloning and expression profiling of the c-Jun N-terminal kinase gene and its response to UV-A stress in *Helicoverpa armigera* (Lepidoptera: Noctuidae). Acta. Entomol. Sin..

[15] Sang W, Ma WH, Qiu L, Zhu ZH, Lei CL (2012). The involvement of heat shock protein and cytochrome P450 genes in response to UV-A exposure in the beetle *Tribolium castaneum*. J. Insect Physiol..

[16] Zhou LJ, Zhu ZH, Liu ZX, Ma WH, Desneux N, Lei CL (2013). Identification and transcriptional profiling of differentially expressed genes associated with response to UV-A radiation in *Drosophila melanogaster* (Diptera: Drosophilidae). Mol. Ecol. Evol..

[17] Wang Z, Gerstein M, Snyder M (2009). RNA-Seq:a revolutionary tool for transcriptomics. Nat. Rev. Genet.

[18] Dettmer K, Aronov PA, Hammock BD (2007). Mass spectrometry-based metabolomics. Mass Spectrom Rev..

[19] Timmermans MJTN, Roelofs D, Nota B, Ylstra B, Holmstrup M (2009). Sugar sweet springtails: On the transcriptional response of *Folsomia candida* (Collembola) to desiccation stress. Insect Mol. Biol..

[20] Xu YJ, Luo FF, Gao Q, Shang YF, Wang CS (2015). Metabolomics reveals insect metabolic responses associated with fungal infection. Anal. Bioanal. Chem..

[21] Macmillan HA (2016). Cold acclimation wholly reorganizes the *drosophila melanogaster* transcriptome and metabolome. Sci. Rep-uk..

[22] Liu YC (2017). Comparative transcriptome analysis of *Glyphodes pyloalis* Walker (Lepidoptera: Pyralidae) reveals novel insights into heat stress tolerance in insects. BMC Genomics.

[23] Sun LL, Liu P, Sun SH, Yan SH, Cao CW (2018). Transcriptomic analysis of interactions between *Hyphantria cunea* larvae and nucleopolyhedrovirus. Pest Manag. Sci..

[24] Zhu W, Meng Q, Zhang H, Wang ML, Zhang JH (2019). Metabolomics reveals the key role of oxygen metabolism in the heat susceptibility of an alpine dwelling ghost moth, *Thitarodes xiaojinensis* (lepidoptera: hepialidae). Insect Sci..

[25] Jing XF, Lei CL (2004). Advances in research on phototaxis of insects and the mechanism. Entomol. Knowl..

[26] Nafus DM, Schreiner IH (1991). Review of the biology and control of the Asian corn borer, *Ostrinia furnacalis* (Lep: Pyralidae). Trop Pest Manag..

[27] Afidchao, M. M., Musters, C. & Snoo, G. R. D. Asian corn borer (ACB) and non-ACB pests in GM corn (*Zea mays* L.) in the Philippines. *Pest Manag. Sci.* **69**, 792–801 (2013). 10.1002/ps.3471 23401215

[28] Guo BQ, Li SW (1997). A study of the rhythmic changes of phototactic behaviour and compound eye structure. Acta. Entomol. Sin..

[29] Yang ZY, Wu WG, Fen HP, Wu W, Guo BQ (1998). The comparison of response characteristics to light stimulation between the compound eyes of cotton bollworm (*Helicoverpa armigera*) and corn borer (*Ostrinia furnacalis*). Acta. Biophys. Sin..

[30] Zhang CY, Meng JY (2018). Identification of differentially expressed proteins in *Ostrinia furnacalis* adults after exposure to ultraviolet A. Environ. Sci. Pollut. Res. Int..

[31] Liu F, Meng JY, Yang CL, Zhang CY (2020). Cloning and expression profile of vitellogenin gene and its response to UV-A stress in *Ostrinia furnacalis* (Guenée). Acta. Entomol. Sin..

[32] Su L, Meng JY, Yang H, Zhang CY (2020). Molecular characterization and expression of OfJNK and Ofp38 in *Ostrinia furnacalis*(Guenée) under different environmental stressors *Front*. Physiol..

[33] Feng CJ (2011). Parasitization by *Macrocentrus cingulum* (Hymenoptera: Braconidae) influences expression of prophenoloxid -ase in Asian corn borer *Ostrinia furnacalis*. Arch. Insect Biochem. Physiol..

[34] Grabherr MG (2011). Full-length transcriptome assembly from RNA-Seq data without a reference genome. Nat. Biotechnol..

[35] Ashburner M (2000). Gene ontology: Tool for the unification of biology. The gene ontology consortium. Fac. Res..

[36] Conesa A, Götz S, García-Gómez JM, Terol J, Talón M, Robles M (2005). Blast2GO: A universal tool for annotation, visualization and analysis in functional genomics research. Bioinformatics.

[37] Li B, Dewey CN (2011). RSEM: Accurate transcript quantification from RNA-Seq data with or without a reference genome. BMC Bioinform..

[38] Mortazavi A, Williams BA, McCue K, Schaeffer L, Wold B (2008). Mapping and quantifying mammalian transcriptomes by RNASeq. Nat. Methods.

[39] Love MI, Huber W, Anders S (2014). Moderated estimation of fold change and dispersion for RNA -seq data with DESeq2. Genome Biol..

[40] Livak KJ, Schmittgen TD (2001). Analysis of relative gene expression data using real-time quantitative PCR and the 2^-ΔΔCT^ method. Methods.

[41] Urbach F (1989). The biological effects of increased ultraviolet radiation: an update. Photochem. Photobiol..

[42] Schauen M, Hornig-Do HT, Schomberg S, Herrmann G, Wiesner RJ (2007). Mitochondrial electron transport chain activity is not involved in ultraviolet A (UV-A)-induced cell death. Free Radical Biol. Med..

[43] Meng JY, Zhang CY, Lei CL (2010). A proteomic analysis of *Helicoverpa armigera* adults after exposure to UV light irradiation. J. Insect physiol..

[44] Yang LT, Lin H, Takahashi Y, Chen F, Walker MA, Civerolo EL (2011). Proteomic analysis of grapevine stem in response to *Xylella fastidiosa* inoculation. Physiol. Mol. Plant Pathol..

[45] Theopold U, Dushay M (2007). Mechanisms of *Drosophila* immunity-an innate immune system at work. Curr. Immunol. Rev..

[46] Ha EM (2005). An antioxidant system required for host protection against gut infection in *Drosophila*. Dev. Cell..

[47] Clavaron-Mathews MC, Summers CB, Felton GW (1997). Ascorbate peroxidase: a novel antioxidant enzyme in insects. Arch. Insect Biochem..

[48] Karthi S, Sankari R, Shivakumar MS (2014). Ultraviolet-B light induced oxidative stress: Effects on antioxidant response of *Spodoptera litura*. J. Photochem. Photobiol. B.

[49] Feyereisen R (2012). Insect CYP genes and P450 enzymes. Insect Mol. Biol. Biochem..

[50] Pappa A, Estey T, Manzer R, Brown D, Vasiliou V (2003). Human aldehyde dehydrogenase 3A1 (ALDH3A1): Biochemical characterization and immunohistochemical localization in the cornea. Biochem. J..

[51] Karunaratne SH, Hemingway J, Jayawardena KG, Vaughan A (1995). Kinetic and molecular differences in the amplified and non-amplified esterases from insecticide-resistant and susceptible *Culex quinquefasciatus* mosquitoes. J. Biol. Chem..

[52] Zhao AP, Zhan EL, Sun C, Liu TX, Li YP (2017). Effects of Cry1Ac toxin on protease and carboxylesterase activities in the larvae midgut of *Plutella xylostella*. J. Plant Prot..

[53] Johnson GL, Lapadat R (2002). Mitogen-activated protein kinase pathways mediated by ERK, JNK, and p38 protein kinases. Science.

[54] Colicelli, J. Human RAS superfamily proteins and related GTPases. *Sci. Signal.* **250**, RE13 (2004). 10.1126/stke.2502004re13 PMC2828947 15367757

[55] Roberts, P, J. & Der, C. J. Targeting the Raf‐MEK‐ERK mitogen‐activated protein kinase cascade for the treatment of cancer. *Oncogene* **26**, 3291–3310 (2007). 10.1038/sj.onc.1210422 17496923

[56] He Y, Ye QL (2003). The Ras radiation resistance pathway of cancer cell. Sci. For. Med. ( Cancer Section).

[57] Ruan MG, Li JQ, Meng SS, Ma J (2015). Cloning and expression profiling in response to low tempe- rature of Ras GTPase-activating protein gene *MpRasGAP* in the desert beetle *Microdera punctipennis* (Coleoptera: Tenebrionidae). Acta. Entomol. Sin..

[58] Wang HH, Lei ZR (2005). Current developments of heat-shock proteins in insect. Sci. Agric. Sin..

[59] Tiedje KE, Stevens K, Barnes S, Weaver DF (2010). Beta-alanine as a small molecule neuro- transmitter. Neurochem. Int..

[60] Kim PM (2010). Aspartate racemase, generating neuronal D-aspartate, regulates adult neurogenesis roc. Natl. Acad. Sci..

[61] Cui SF, Wang L, Qiu JP, Liu ZC, Geng XQ (2017). Comparative metabolomics analysis of *Callosobruchus chinensis* larvae under hypoxia, hypoxia/hypercapnia and normoxia. Pest Manag. Sci..

[62] Wu G (2013). Functional amino acids in nutrition and health. Amino Acids.

[63] Clark, T. C., Tinsley, J., Sigholt, T., Macqueen, D. J. & Martin, S. A. M. Supplementation of arginine, ornithine and citrulline in rainbow trout (*Oncorhynchus mykiss*): Effects on growth, amino acid levels in plasma and gene expression responses in liver tissue. *Comp. Biochem. Physiol. Part A Mol. Integr. Physiol.* **241**, 110632–110632 (2019). 10.1016/j.cbpa.2019.110632 31812671

[64] Zorov DB, Juhaszova M, Sollott SJ (2006). Mitochondrial ROS-induced ROS release: An update and review. Biochim. Biophys. Acta. Bioenerg..

[65] Rowe L, Degtyareva N, Doetsch P (2008). DNA damage-induced reactive oxygen species (ROS) stress response in *Saccharomyces cerevisiae*. Free Radical Biol. Med..

[66] Hashimoto M (2002). Docosahexaenoic acid provides protection from impairment of learning ability in Alzheimer’s disease model rats. J. Neurochem..

[67] Bazan NG (2008). Neuroprotectin D1-mediated anti-inflammatory and survival signaling in stroke, retinal degenerations, and Alzheimer’s disease. J. Lipid Res..

[68] Wu A, Noble EE, Tyagi E, Ying Z, Zhuang Y, Gomez-Pinilla F (2015). Curcumin boosts DHA in the brain: implications for the prevention of anxiety disorders. Biochim. Biophys. Acta Mol. Basis Dis..

[69] Tallima H, Ridi RE (2018). Arachidonic acid: Physiological roles and potential health benefits—A review. J. Adv. Res..

[70] Nayak S, Pradhan S, Sahoo D, De Parida AD (2020). novo transcriptome assembly and analysis of *Phragmites karka*, an invasive halophyte, to study the mechanism of salinity stress tolerance. Sci. Rep..

[71] Teng Q, Huang W, Collette TW, Ekman DR, Tan C (2009). A direct cell quenching method for cell-culture based metabolomics. Metabolomics.

[72] Knee, J. M., Rzezniczak, T. Z., Barsch, A., Guo, K. Z. & Merritt, T. J. S. A novel ion pairing LC/MS metabolomics protocol for study of a variety of biologically relevant polar metabolites. *J. Chromatogr. B. Anal. Technol. Biomed. Life Sci.* **936**, 63–73 (2013). 10.1016/j.jchromb.2013.07.027 24004912

[73] Zhang CY, Meng JY, Yang KX (2017). Population dynamics of light-trap collection of *Ostrinia furnacalis* in Guizhou Province. Guizhou Agric. Sci..

[74] Sang W, Huang QY, Wang XP, Guo SH, Lei CL (2019). Progress in research on insect phototaxis and future prospects for pest light-trap technology in China. Chin. J. Appl. Entomol..

[75] Grubor-Lajsic G, Block W, Worland R (1992). Comparison of the cold hardiness of two larval Lepidoptera (Noctuidae). Physiol. Entomol..

[76] Bale JS, Hayward SAL (2010). Insect overwintering in a changing climate. J. Exp. Biol..

[77] Thomas MB, Blanford S (2003). Thermal biology in insect-parasite interactions. Trends Ecol. Evol..

[78] Sinclair BJ, Ferguson LV, Salehipour-shirazi G, MacMillan HA (2013). Cross-tolerance and cross-talk in the cold: Relating low temperatures to desiccation and immune stress in insects. Integr. Comp. Biol..

[79] Wojda I (2017). Temperature stress and insect immunity. J. Therm. Biol..

[80] Wang Y, Wang LJ, Zhu ZH, Ma WH, Lei CL (2012). The molecular characterization of antioxidant enzyme genes in *Helicoverpa armigera* adults and their involvement in response to ultraviolet-A stress. J. Insect Physiol..

[81] Liu XF, Meng JY, Zhang Y, Zhang CY (2018). Effects of UV-B radiation on the antioxidant system of *Myzus persicae*. J. Environ. Entomol..

[82] Li Q (2012). Effects of temperature stress and ultraviolet radiation stress on antioxidant systems of *Locusta migratoria tibetensis* Chen. Acta Ecol. Sin..

[83] Jin, T. T., Xue, C., Angharad, M. R. G., Wang, Z. Y., Martin., G. E & He, K. L. Down regulation and mutation of a cadherin gene associated with Cry1Ac resistance in the Asian corn borer *Ostrinia furnacalis* (Guenée). *Toxins* **6**, 2676–2693 (2014). 10.3390/toxins6092676 PMC4179154 25216082

[84] Zhang ZK, Lu Y, Xu WJ, Du Q, Sui L, Zhao Y, Li QY (2019). RNA sequencing analysis of *Beauveria bassiana* isolated from *Ostrinia furnacalis* identifies the pathogenic genes. Microb. Pathog..

[85] Roy S, Saha TT, Zou Z, Raikhel AS (2018). Regulatory pathways controlling insect reproduction. Annu. Rev. Entomol..

[86] Riddiford, L. M. Cellular and Molecular Actions of juvenile kormone I. General considerations and premetamorphic actions. *Adv. Insect Physiol.* **24**, 213–274 (1994).

[87] Wyatt, G. R., & Davey, K. G. Cellular and molecular actions of juvenile hormone. II. Roles of juvenile hormone in adult insects. *Adv. Insect Physiol.* **26**, 1–155 (1996).

[88] Piao, Z. F, Ui-Tei, K., Nagano, M. & Miyata, Y. Participation of intracellular Ca^2+^/calmodulin and protein kinase(s) in the pathway of apoptosis induced by a *Drosophila* cell death gene, reaper. *Mol. Cell Biol. Res. Commun.* **4**, 307–312 (2001). 10.1006/mcbr.2001.0297 11529681

[89] Mcphalen CA, Strynadka NC, James MN (1991). Calcium-binding sites in proteins: A structural perspective. Adv. Protein. Chem. Struct..

[90] Arakane Y (2011). Both UDP N-acetylglucosamine pyrophosphorylases of *Tribolium castaneum* are critical for molting, survival and fecundity. Insect Biochem. Mol. Biol..

[91] Lee CH, Sidikd K, Chin KV (2001). Role of cAMP-dependent protein kinase in the regulation of DNA repair. Cancer Lett..

[92] Sang W, Zhu ZH, Lei CL (2016). Review of phototaxis in insects and an introduction to the light stress hypothesis. Chin. J. Appl. Entomol..

