# Supplementary material for: Comparative transcriptome and metabolome analysis of Ostrinia furnacalis female adults under UV-A exposure
Source: Sci Rep. 2021 Mar 24;11:6797. doi: 10.1038/s41598-021-86269-0 (PMC7990960; doi:10.1038/s41598-021-86269-0)
Supplement: Supplementary file 1 — Supplementary Information. [file 41598_2021_86269_MOESM1_ESM.pdf]

Support information

Comparative transcriptome and metabolome analysis of *Ostrinia furnacalis* female adults under UV-A exposure

Li Su[1], ChangLi Yang[1], JianYu Meng[2], Lv Zhou[1], ChangYu Zhang[1*]

Figure S1. Gene homology searches against the Nr database. (a) Similarity distribution of the highest BLAST hits per unigene. (b) The *e*-value distribution of BLAST hits per unigene with a cut-off of 0. (c) Species classification displayed as a percentage of all homologous gene hits. The top four hits belong to Lepidoptera. BLAST, Basic Local Alignment Search Tool.

Figure S2. GO categories analysis of DEGs between UV exposure (UV1h and UV2h) and control group (CK) in *Ostrinia furnacalis*. Pink represents "biological process," green represents "cellular component," and blue represents "molecular function." GO, Gene Ontology; UV, ultraviolet; DEGs, differentially expressed genes.

Figure S3. PLS-DA score plots of UV-A-treated and control groups. POS ion mode: UV1h vs. CK (A), UV2h vs. CK (B), and UV2h vs. UV1h (C). NEG ion mode: UV1h vs. CK (D), UV2h vs. CK (E), and UV2h vs. UV1h (F). PLS-DA, partial least squares–discriminant analysis; UV, ultraviolet; POS, positive; NEG, negative.

Figure S4. Permutation tests ($n = 200$) of projections to PLS-DA models for comparative analysis of three groups (UV1h vs. CK, UV2h vs. CK, and UV2h vs. UV1h) in POS (A–C) and NEG (D–F) ion modes. Blue dots and red squares represent R^2 and Q^2 values, respectively, obtained through permutation tests, and dashed lines represent corresponding regression lines. R^2, coefficient; Q^2, cross-validated correlation coefficient; PLS-DA, partial least squares–discriminant analysis; UV, ultraviolet; POS, positive; NEG, negative.

Figure S5. Volcano plots of DEMs identified in three comparative analyses. POS ion mode: UV1h vs. CK (A), UV2h vs. CK (B), and UV2h vs. UV1h (C). NEG ion mode: UV1h vs. CK (D), UV2h vs. CK (E), and UV2h vs. UV1h (F). The dot size represents the VIP value of each DEM. Red and green dots represent upregulated and downregulated DEMs, respectively, and gray dots indicate that the metabolites had insignificant differences (NoDiff) in the comparative analysis. DEMs, differentially expressed metabolites; UV, ultraviolet; POS, positive; NEG, negative; VIP, variable importance in the projection.

Table S1. Primers used for qPCR analysis.

Table S2. Length distribution of unigenes in the *Ostrinia furnacalis* transcriptome.

Table S3. Functional annotation result of *Ostrinia furnacalis.*

Table S4. All significantly changed KEGG pathways in UV1h compared to CK. KEGG, Kyoto Encyclopedia of Genes and Genomes; UV, ultraviolet.

Table S5. All significantly changed KEGG pathways in UV2h compared to CK. KEGG, Kyoto Encyclopedia of Genes and Genomes; UV, ultraviolet.

Table S6. Metabolites significantly changed in *Ostrinia furnacalis* in UV1h compared to CK. UV, ultraviolet.

Table S7. Metabolites significantly changed in *Ostrinia furnacalis* in UV2h compared to CK. UV, ultraviolet.

Table S8. Metabolites significantly changed in *Ostrinia furnacalis* in UV2h compared to UV1h. UV, ultraviolet.

Table S9. KEGG enrichment analysis of the differential metabolic pathway in *Ostrinia furnacalis* in UV1h compared to CK. KEGG, Kyoto Encyclopedia of Genes and Genomes; UV, ultraviolet.

Table S10. KEGG enrichment analysis of the differential metabolic pathway in *Ostrinia furnacalis* in UV2h compared to CK. KEGG, Kyoto Encyclopedia of Genes and Genomes; UV, ultraviolet.

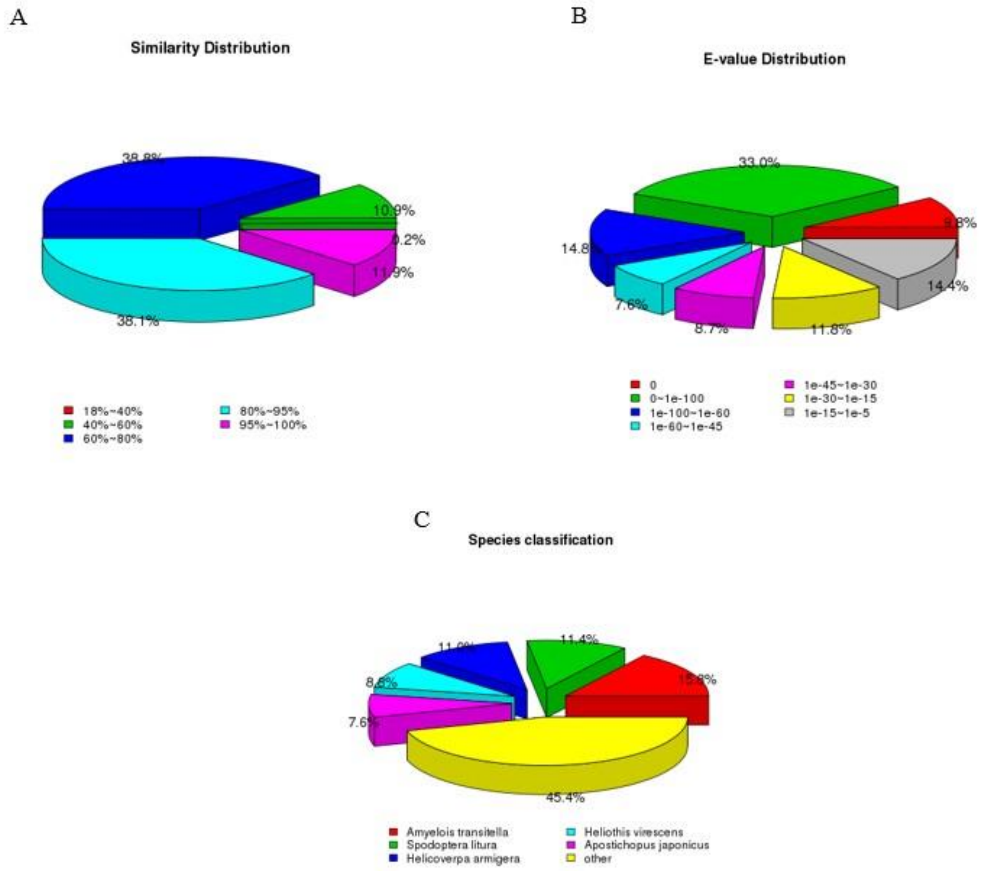

Figure S1. Gene homology searches against the Nr database. (a) Similarity distribution of the highest BLAST hits per unigene. (b) The *e*-value distribution of BLAST hits per unigene with a cut-off of 0. (c) Species classification displayed as a percentage of all homologous gene hits. The top four hits belong to Lepidoptera. BLAST, Basic Local Alignment Search Tool.

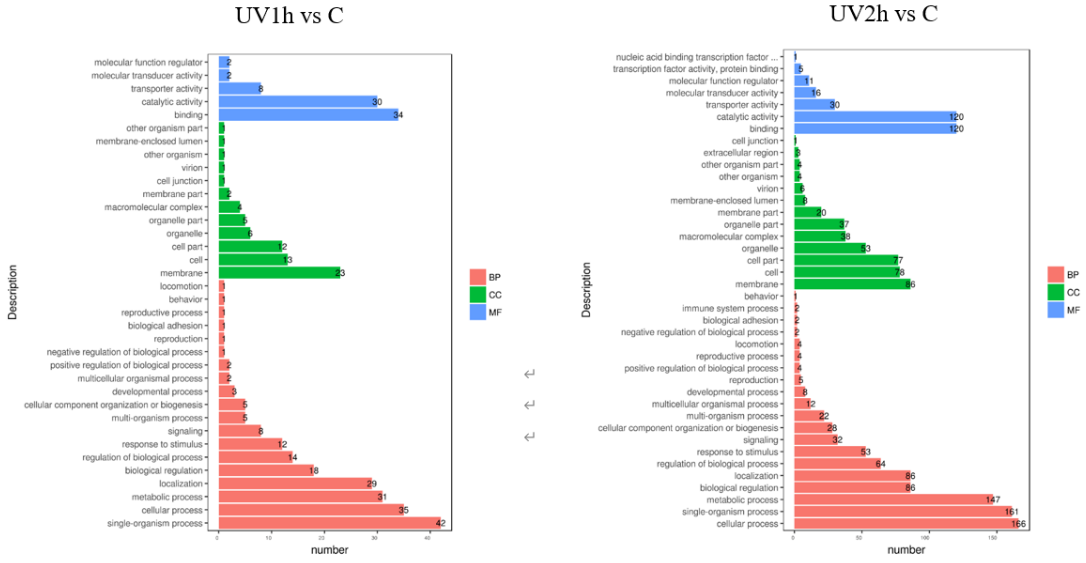

Figure S2. GO categories analysis of DEGs between UV exposure (UV1h and UV2h) and control group (CK) in *Ostrinia furnacalis*. Pink represents "biological process," green represents "cellular component," and blue represents "molecular function." GO, Gene Ontology; UV, ultraviolet; DEGs, differentially expressed genes.

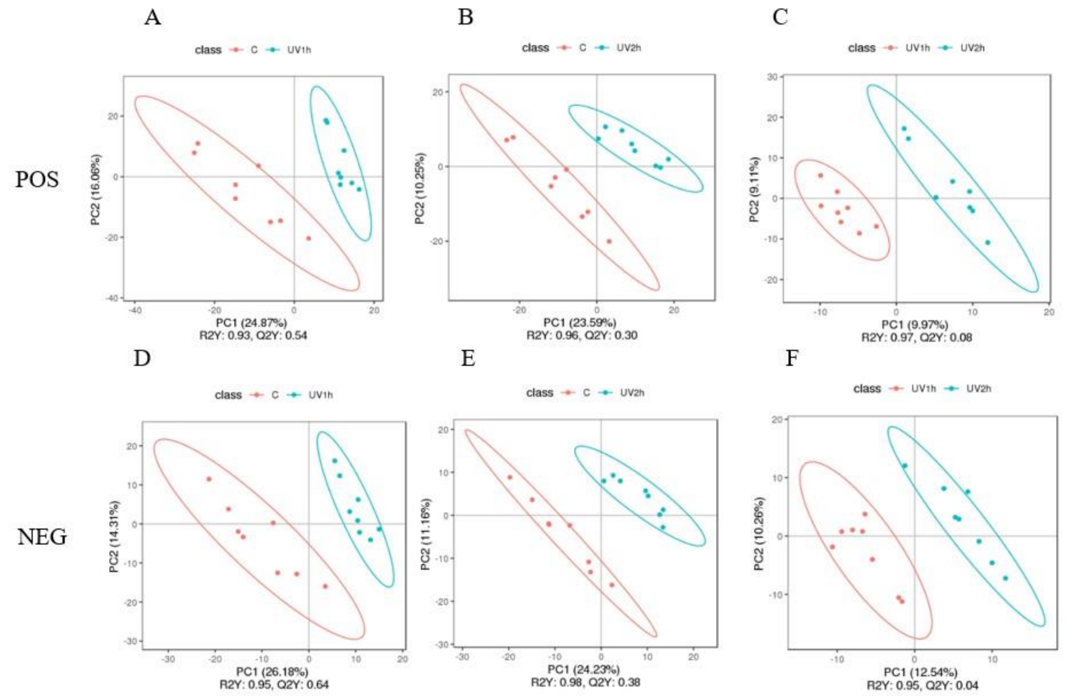

Figure S3. PLS-DA score plots of UV-A-treated and control groups. POS ion mode: UV1h vs. CK (A), UV2h vs. CK (B), and UV2h vs. UV1h (C). NEG ion mode: UV1h vs. CK (D), UV2h vs. CK (E), and UV2h vs. UV1h (F). PLS-DA, partial least squares–discriminant analysis; UV, ultraviolet; POS, positive; NEG, negative.

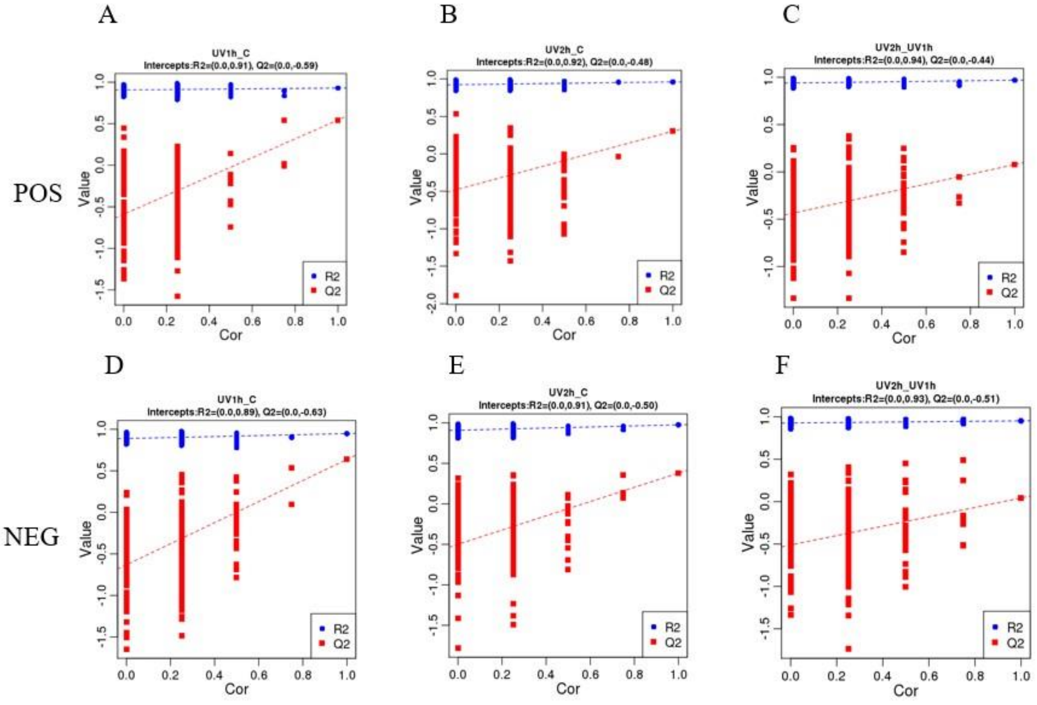

FigureS4. Permutation tests (n = 200) of the projections to latent structures-discriminant analysis (PLS-DA) models for the comparative analysis of the three groups (UV1h vs. C, UV2h vs. C, and UV2h vs. UV1h) in the positive (POS: A,B,C) and negative (NEG:D,E,F) detection modes. The blue dots and the red squares express the R^2 and Q^2 values, respectively, obtained through the permutation tests, and the dashed lines represent corresponding regression lines. R^2: coefficient; Q^2: cross-validated correlation coefficient.

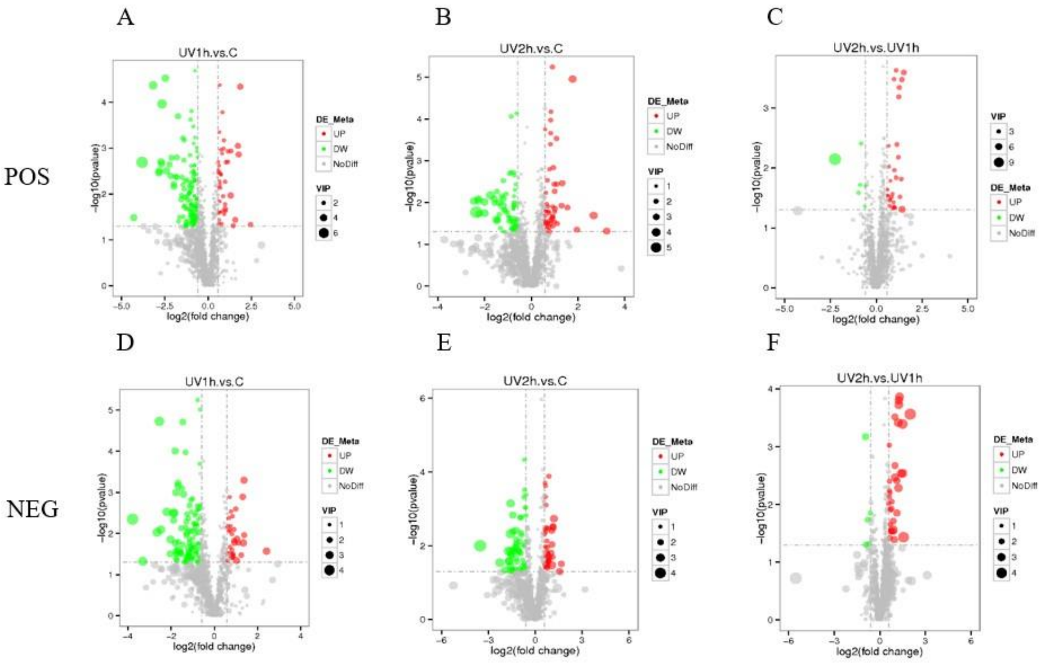

Figure S5. Volcano plots of DEMs identified in three comparative analyses. POS ion mode: UV1h vs. CK (A), UV2h vs. CK (B), and UV2h vs. UV1h (C). NEG ion mode: UV1h vs. CK (D), UV2h vs. CK (E), and UV2h vs. UV1h (F). The dot size represents the VIP value of each DEM. Red and green dots represent upregulated and downregulated DEMs, respectively, and gray dots indicate that the metabolites had insignificant differences (NoDiff) in the comparative analysis. DEMs, differentially expressed metabolites; UV, ultraviolet; POS, positive; NEG, negative; VIP, variable importance in the projection.

Table S1. Primers used for qPCR analysis.

**Gene number**	**Forward primer (5′-3′)**	**Reverse primer (5′-3′)**
Cluster-7827.26019	ACCGCCGCTTTAAACGTGA	TGGACCACCCAACACCTTCT
Cluster-7827.7550	CGCTTCTCATGGGCGACTAC	AGCCGAGCGCTATAGGAACT
Cluster-7827.19564	AATGGAGTCGTGTCTTCGCT	AGCCTGTCACAAGGATCCAC
Cluster-7827.9082	AAACACGTCACCGAACCAGA	ATCAGCCCACGTGTTGTAGT
Cluster-7827.24093	TAACCTTGGACCTGACGCAC	CACTGCGATCTTCCCACTCA
Cluster-7827.14559	GCTTTTCAAGGAACCGCCAA	CAAAGCATAGAGACGCACAGC
Cluster-7827.14319	TGTACTACGCACGAGATGGTT	CCCCAATTCCAGGGGATACAG
Cluster-7827.14821	ACGCTGTTACCAAAGGCGAG	ATCCTCTCGGGCTCGTAGTCG
Cluster-7827.5951	ACACTTTGACTCACCTCGTCC	CGGCGACAACAAGGGAGAA
Cluster-7827.27454	TTGATGAAGAAGCAAGAGTGGA	ACTCGTATGGGTGTGGGAAT
Cluster-7827.28504	TGGAAGCAGCGAGATACATCA	GCGATCATGCCGTCATAGTTC
Cluster-7827.4700	AGTGGAAGATGGCGGATTGTA	GGTGGCAGAGTCCTGATGTAT
Cluster-7827.1116	ACAACAGATGTGGTGTGGAATT	CCTCTTGCCGTTCATACCTTC
Cluster-7827.3726	GAGAACCTTCCTTGGCAGAAC	ATGATGGAGTCGGCGAGTG
Cluster-7827.7896	CGCTGCACACGCTAATACTG	TCGGTCAGCAGGTTCTCTTC
β-actin	TACGAAGGTTACGCTCTGCC	ATGTCACGCACGATTTCCCT
GAPDH	TTGAGGTTCAGGGTGACAAGG	CACCTTCCAAGTGAGCCGA

Table S2. Length distribution of the unigenes in the *O. furnacalis* transcriptome.

Length of unigenes	Total number	Percentage (%)
200-500	19317	38.55
500-1000	14376	28.69
1000-2000	7712	15.39
≥2000	8701	17.37
All unigenes	50106	
Length of all unigenes (nt)	62494111	
N50 (bp)	2299	

Table S3. Functional annotation result of *O. furnacalis*.

Database	Number of Genes	Percentage (%)
Annotated in NR	19207	38.33%
Annotated in NT	20888	41.68%
Annotated in Swiss-Prot	12115	24.17%
Annotated in GO	14563	29.06%
Annotated in KEGG	7221	14.41%
Annotated in KOG	7028	14.02%
Annotated in all Databases	3484	6.95%
Annotated in at least one Database	31279	62.42%
Total Unigenes	50106	100

Table S4. All the significantly changed KEGG pathways in UV1h compare to CK.

**Pathway**	**DEGs with pathway annotation**	**All genes with pathway annotation**	**P-value**	**Pathway ID**
ABC transporters	4	48	0.000257497	ko02010
Amyotrophic lateral sclerosis (ALS)	2	32	0.017448588	ko05014
Phototransduction - fly	2	42	0.028316911	ko04745
Inflammatory mediator regulation of TRP channels	2	52	0.041231501	ko04750
Bile secretion	2	54	0.044035867	ko04976
GnRH signaling pathway	2	57	0.048371126	ko04912

Table S5. All the significantly changed KEGG pathways in UV2h compare to CK.

**Pathway**	**DEGs with pathway annotation**	**All genes with pathway annotation**	**P-value**	**Pathway ID**
Bile secretion	7	54	0.001455914	ko04976
ABC transporters	6	48	0.003716946	ko02010
ECM-receptor interaction	6	50	0.00446106	ko04512
Focal adhesion	11	149	0.005130763	ko04510
Vitamin digestion and absorption	3	15	0.012743522	ko04977
Aldosterone-regulated sodium reabsorption	3	20	0.024975175	ko04960
Proximal tubule bicarbonate reclamation	3	22	0.031145059	ko04964
Fatty acid elongation	4	40	0.033296222	ko00062
Cell adhesion molecules (CAMs)	3	25	0.041750883	ko04514
AGE-RAGE signaling pathway in diabetic complications	4	46	0.049702072	ko04933

Table S6. Metabolites significantly changed in *Ostrinia furnacalis* in UV1h compared to CK.

	Metabolite name	RT (min)	Regulated	VIP	P-value	Fold change
sugars	Glucose	1.356	down	3.1276	0.0002	0.6256
	D-glucopyranoside	11.088	down	1.7418	0.0086	0.4505
	D-xylopyranoside	9.241	down	1.0945	0.0425	0.4775
	D-erythro-pentopyranose	9.677	down	1.0152	0.0498	0.3824
	Glucuronamide	2.672	down	1.8000	0.0061	0.2529
	2-Deoxypentose	5.637	down	1.5494	0.0109	0.5745
	N-Acetyl-D-glucosaminitol	6.848	down	1.0941	0.0378	0.4263
amino acid	L-serine	15.411	up	1.2373	0.0213	1.6524
	Phosphoarginine	1.259	up	2.6197	0.0267	5.3501
	L-Aspartic acid	1.284	up	2.4527	0.0452	2.0387
	Cysteinylglycine	1.81	up	1.1924	0.0034	1.6006
	DL-tyrosine	7.11	up	1.8175	0.0235	1.9963
	DL-Arginine	1.269	up	1.2057	0.0439	1.5374
	Acetyl-L-methionine	3.584	down	3.4178	0.000019	0.3625
	N-Acetylornithine	1.424	down	4.5087	5.67E-06	0.5849
	L-(+)-Citrulline	1.382	down	1.0820	0.0383	0.5324
	D-tryptophan	10.546	down	0.0108	0.3474	1.4505
fatty acid	Arachidonic acid	12.664	up	3.0992	4.60E-05	3.6273
	Ethyl Linoleate	13.155	up	1.9009	0.0012	2.2256
	Leukotriene D4	15.429	up	1.3230	0.0013	1.7626
	2-Arachidonoyl glycerol	15.098	up	1.0175	0.0030	1.5125
	Docosahexaenoic acid	14.604	up	1.1056	0.0261	1.5201
	Tetradecanedioic acid	10.908	down	1.9281	0.0057	0.5147
	Dodecanedioic acid	9.409	down	1.9202	0.0059	0.5373
	Azelaic acid	5.195	down	1.8181	0.0078	0.5272
organi	Phenylglyoxylic acid	7.1	up	2.1756	0.0012	2.5038

c acid	3-Methyladipic acid	1.619	down	1.2064	0.0341	0.5911
	2-Hydroxyglutaric acid	2.395	down	1.0785	0.0404	0.5986
others	Redul	6.83	down	3.4875	1.88E-05	0.1712
	Carbamazepine	7.915	down	1.4948	0.0138	0.4363
	Rutaevin	2.133	down	1.3904	0.0268	0.2953

Table S7. Metabolites significantly changed in *Ostrinia furnacalis* in UV2h compared to CK.

	Metabolite name	RT (min)	Regulated	VIP	P-value	Fold change
sugars	Uridine 5'-diphosphogalactose	1.271	up	1.1562	0.0005	1.5792
	UDP-N-acetylglucosamine	1.339	up	1.1941	0.0156	1.7592
	D-glucopyranoside	12.084	up	1.6493	0.0249	1.8272
	N-Acetyl-D-glucosaminitol	6.848	down	1.4599	0.0498	0.4427
	Glucose	1.356	down	1.2570	0.0003	0.6211
	2-Deoxypentose	5.637	down	1.2572	0.0142	0.5906
amino acid	N-Acetylleucylleucine	8.558	up	1.5468	0.0289	1.9969
	D-methioninamide	9.348	down	2.2657	0.0252	0.3705
	Acetyl-L-methionine	3.584	down	1.8945	0.0017	0.5186
	Phosphatidylserine	5.792	down	2.8261	0.0279	0.3608
	L-alanine	5.852	down	1.4955	0.0405	0.5889
fatty acid	Arachidonic acid	12.664	up	3.4325	0.0001	3.3988
	Leukotriene D4	15.429	up	1.6304	0.0002	1.7751
	Adipic acid	1.482	up	2.8672	0.0336	2.0781
	butanoate	13.866	down	1.5128	0.0022	0.5980
	hexadecandioic acid	13.022	down	2.1139	0.0147	0.4530
	3-Methyladipic acid	1.619	down	1.2954	0.0245	0.5744
	Azelaic acid	5.195	down	1.0422	0.0305	0.6039
	Dodecanedioic acid	9.409	down	1.1307	0.0346	0.6391
organic acid	Benzoic acid	15.428	up	1.6903	0.0001	1.7721
	Citric acid	1.314	up	1.0778	0.0301	1.6147
	Phenylglyoxylic acid	7.1	up	1.6372	0.0166	1.9865

	Anthranilic acid	8.029	down	3.3981	0.0097	0.2484
	4-Phenolsulfonic acid	5.483	down	2.7271	0.0263	0.3135
	Anthranilic acid	3.014	down	3.0266	0.0288	0.2077
others	Velban	12.938	up	1.5715	0.0179	1.6882
	Zileuton	8.493	down	1.4948	0.0138	3.1768
	Tolcapone	8.454	down	2.6748	0.0263	0.3091

Table S8. Metabolites significantly changed in *Ostrinia furnacalis* in UV2h compared to UV1h.

	Metabolite name	RT (min)	Regulate d	VIP	P-value	Fold change
sugars	3-Phosphoglyceric acid	1.284	up	2.0143	0.0294	1.6791
amino acid	Tryptoline	6.64	up	3.5558	0.0157	2.3453
	Thyronine	9.961	up	2.9011	0.0001	2.3868
	Gamma-L-glutamyl-L-tyrosine	1.406	up	1.4464	0.0058	1.5457
	Metirosine	6.248	down	1.6054	0.0477	0.6187
fatty acid	(R)-3-hydroxybutyrylcarnitine	1.392	up	2.2657	0.0351	1.6657
	Ethyl Linoleate	13.155	down	1.6209	0.0436	0.6451
organic acid	D-a-Hydroxyglutaric acid acid	1.3	up	1.5559	0.0221	1.6106
	(.+/-.)- Tartaric acid	1.221	up	3.4765	0.0276	1.8136
	Phthalic acid	1.839	up	1.7237	0.0089	1.7284

Values were means of duplicate analysis. The partial least squares–discriminant analysis (PLS-DA) model of variable importance in the projection (VIP) was used for screening significant differences between two groups (VIP > 1).Intergroup nonparametric Tukey's tests were performed (P < 0.05); the lower the value, the more reliable the result.

Table S9. KEGG enrichment analysis of the differential metabolic pathway in *Ostrinia furnacalis* in UV1h compared to CK.

Pathway name	Total Metabolite number	Mapping Metabolite number	MapID
Biosynthesis of amino acids	20	5	map01230
Histidine metabolism	14	4	map00340
Glycine, serine and threonine metabolism	10	3	map00260
Nicotinate and nicotinamide metabolism	10	4	map00760
Arginine biosynthesis	5	3	map00230
Alanine, aspartate and glutamate metabolism	12	3	map00250
Tryptophan metabolism	10	2	map00380
Fc epsilon RI signaling pathway	3	3	map04664
Arginine and proline metabolism	6	2	map00330
2-Oxocarboxylic acid metabolism	4	2	map01210
Arachidonic acid metabolism	4	3	map01120
Citrate cycle (TCA cycle)	6	2	map00020
Pantothenate and CoA biosynthesis	6	2	map00770
Glyoxylate and dicarboxylate metabolism	11	2	map00630
Glycerophospholipid metabolism	5	3	map00564
Lysine biosynthesis	3	2	map00300
Phenylalanine, tyrosine and tryptophan biosynthesis	6	1	map00400
beta-Alanine metabolism	6	2	map00410
Aminoacyl-tRNA biosynthesis	7	1	map00970
Cysteine and methionine metabolism	2	1	map00270
Tyrosine metabolism	8	1	map00350

Table S10. KEGG enrichment analysis of the differential metabolic pathway in *Ostrinia furnacalis* in UV2h compared to CK.

Pathway name	Total Metabolite number	Mapping Metabolite number	MapID
Arachidonic acid metabolism	20	5	map00590
Glycerophospholipid metabolism	9	2	map00564
Glycine, serine and threonine metabolism	3	2	map00260
Phenylalanine metabolism	2	2	map00360
Tryptophan metabolism	6	2	map00380
Linoleic acid metabolism	3	1	map00591
Protein digestion and absorption	9	1	map04974
Tyrosine metabolism	11	2	map00350
Arginine and proline metabolism	7	2	map00330
Phenylalanine, tyrosine and tryptophan biosynthesis	5	1	map00400
Fc epsilon RI signaling pathway	3	3	map04664
Synaptic vesicle cycle	4	2	map04721
Amino sugar and nucleotide sugar metabolism	2	1	map00520
Pantothenate and CoA biosynthesis	2	1	map00770
Ascorbate and aldarate metabolism	2	1	map00053
beta-Alanine metabolism	1	1	map00410
Nicotinate and nicotinamide metabolism	1	1	map00760
Histidine metabolism	1	1	map00340
Glutathione metabolism	1	1	map00480
